# Targeting Tris(2,3-dibromopropyl) Isocyanurate-Induced Inflammation in Hippocampal Neurons In Vitro: Mechanistic Insights and Implications for Neurodegenerative Disease Prevention

**DOI:** 10.1007/s12035-025-05301-w

**Published:** 2025-11-19

**Authors:** Dominika Szlachcikowska, Oliwia Koszła, Przemysław Sołek, Anna Tabęcka-Łonczyńska

**Affiliations:** 1 Department of Biotechnology and Cell Biology, Medical College, University of Information Technology and Management in Rzeszow, Sucharskiego 2, 35-225 Rzeszow, Poland; 2 Department of Biopharmacy, Medical University of Lublin, Chodźki 4a, 20-093 Lublin, Poland; 3 Department of Biochemistry and Toxicology, University of Life Sciences, Akademicka 13, 20-950 Lublin, Poland

**Keywords:** TBC, Hippocampal cells, Inflammation, Caspase activity, NF-κB

## Abstract

Tris(2,3-dibromopropyl) isocyanurate (TBC) represents an emerging environmental contaminant with potential neurotoxic effects, attributable to its tendency to bioaccumulate and its capacity to traverse the blood–brain barrier. This study investigates the impact of TBC on cellular metabolism, membrane integrity or apoptosis-related caspase activity in mouse hippocampal cells, examining its interaction with key signaling pathways. Our results indicated that while TBC did not exhibit significant cytotoxicity at lower concentrations, prolonged exposure to higher concentrations substantially decreased metabolic activity and increased apoptotic marker activities (caspase-1, -3 and -9). Co-treatment with CAY10464 (AhR antagonist), GW9662 (PPARγ antagonist) and honokiol (NF-κB inhibitor) revealed that TBC significantly modulated NF-κB signaling, with notable reductions in AhR and IκBα protein expressions and altered levels of mTOR, NF-κB and p-IκBα proteins. Furthermore, TBC induced calcium release, supporting its role in neuroinflammatory responses. Moreover, TBC inhibited antioxidant enzyme activity and increased proinflammatory cytokine expression, implicating oxidative stress and inflammation in its neurotoxic effects. Antioxidants and Keap1-Nrf2 pathway inhibitors partially attenuated these responses, highlighting the role of oxidative stress pathways. These results suggest that prolonged TBC exposure may impair neuronal metabolism and activate apoptotic pathways, with NF-κB playing a pivotal role in mediating its neurotoxic effects.

**Graphical Abstract:**

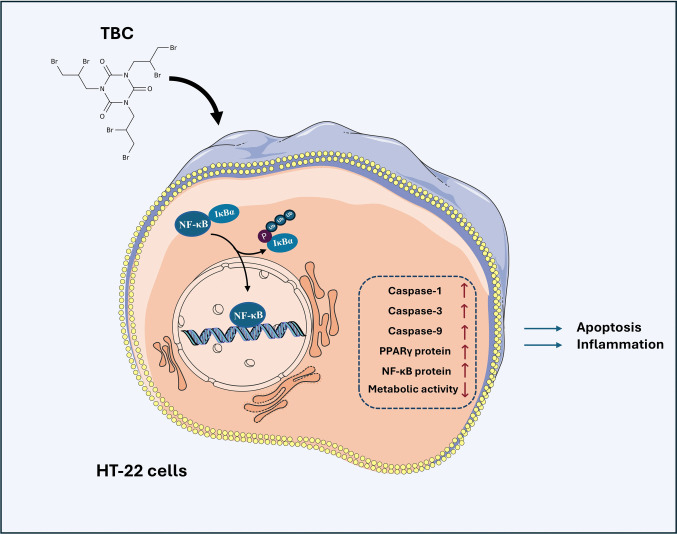

**Supplementary Information:**

The online version contains supplementary material available at 10.1007/s12035-025-05301-w.

## Introduction

Novel brominated flame retardants (NBFRs) represent new integrated components into a diverse array of consumer products, while their widespread use has resulted in significant environmental contamination [1]. NBFRs are released into the environment through emissions during production, degradation of products at high temperatures and the breakdown of photodegraded substances [2–4]. They have been detected at substantial concentrations across various environmental matrices, including the atmosphere, soil, ground, water and sediments [5, 6]. Despite this fact, the full extent of their potential risks remains inadequately explored, particularly regarding their long-term impact on environmental and human health.

NBFRs, characterized by their persistence and bioaccumulative properties, pose emerging challenges as environmental contaminants. They have been shown to accumulate in biota, exhibit biological toxicity, form enduring residues and undergo long-range atmospheric transport [7–10].

Toxicological studies have identified adverse impacts, including neurotoxicity, hormonal disruptions and endocrine disturbances [11–13]. Among these, tris(2,3-dibromopropyl) isocyanurate (TBC) emerges as a compound of particular concern due to its high toxicity, environmental persistence and capacity to cross the blood–brain barrier [14, 15]. Especially, TBC has been implicated with neuronal toxicity, leading to significant cognitive impairments [16–18] or inducing apoptosis in primary neocortical astrocytes through inflammation [19, 20]. These findings suggest that TBC may contribute to the pathogenesis of neurodegenerative diseases, underscoring the necessity for a more comprehensive investigation into its underlying mechanisms of action.

The selection of exposure doses in this study was informed by environmental concentrations and bioaccumulation data. TBC has been detected at nanogram-per-liter levels in water and microgram-per-kilogram levels in sediments, with bioaccumulation factors up to 10^5^ in aquatic organisms, indicating the potential for significant magnification through the food chain [21, 22]. Prior studies have demonstrated neurotoxic effects at micromolar concentrations in vitro, supporting the use of doses spanning environmentally relevant and higher concentrations [19]. These levels mimic plausible exposures in neuronal tissues, accounting for TBC’s capacity to cross the blood–brain barrier and bioaccumulate [23]. This approach ensures the experimental model reflects realistic scenarios for assessing neurotoxic and neuroinflammatory mechanisms.

The peroxisome proliferator-activated receptor gamma (PPARγ) is a key regulator of cellular homeostasis, playing a critical role in antioxidant defense and detoxification processes across various tissues, including the nervous system [24, 25]. PPARγ modulates the expression of mRNA for antioxidant enzymes such as catalase, superoxide dismutases (SOD1 and SOD2) and glutathione peroxidase (GPx), as well as genes involved in anti-inflammatory responses [26]. Additionally, PPARγ influences the expression of the aromatic hydrocarbon receptor (AhR), a xenobiotic-responsive transcription factor acting as a cellular biosensor [27, 28]. Crosstalk between PPARγ and AhR pathways has been documented, whereby agonists or antagonists of one receptor can modulate the activity of the other [29]. Based on previous evidence implicating AhR in the effects of brominated flame retardants [21] and the anti-inflammatory and neuroprotective roles of PPARγ, including responses to TBBPA [30], we focused on these pathways as potential mediators of TBC-induced effects in hippocampal cells.

Also, the NF-κB signaling pathway, a key mediator of inflammation, is activated by various stimuli and plays a crucial role in the cellular response to damage. This pathway’s involvement in both neuroprotection and neurodegeneration highlights its significance in understanding the toxic effects of TBC and developing potential therapeutic strategies [31, 32].

Honokiol, a natural compound derived from *Magnolia grandiflora* and *Magnolia dealbata*, is recognized as a nuclear factor kappa light chain enhancer of activated B cells (NF-κB) inhibitor and a non-adipogenic PPARγ agonist [33–35]. Notably, honokiol has been shown to cross the blood–brain barrier and exert effects on astrocytes in mouse models [36]. Evidence suggests that honokiol possesses therapeutic potential in neurodegenerative diseases and can mitigate several pathological conditions through its anti-inflammatory, anti-angiogenic, anti-tumor, antioxidant and neuroprotective properties [36].

This study employs the mouse hippocampal cell line to investigate the effects of TBC on neuroinflammatory and neurotoxic pathways. Through a detailed analysis of TBC’s impact on metabolic activity, cellular stress indicators and the expression of critical proteins associated with inflammation and toxicity, this research seeks to elucidate the underlying mechanisms by which TBC contributes to neurodegenerative processes. The findings of this study are crucial for advancing the understanding of the environmental risks associated with novel brominated flame retardants and for informing future risk assessment and management strategies aimed at mitigating their impact on human health and the environment.

## Materials and Methods

### Reagents

The study utilized a variety of reagents and materials sourced from reputable suppliers. Dulbecco’s Modified Eagle Medium (DMEM) without phenol red and phosphate-buffered saline (PBS) without Ca^2+^ and Mg^2+^ were procured from Corning (USA). Fetal bovine serum (FBS) and the Perfect Tricolor Protein Ladder were obtained from EURx (Poland). Essential chemicals and reagents, including trypsin, penicillin, streptomycin, ethanol, methanol, Tris–HCl, Tris-Base, glycine, acrylamide/bisacrylamide, sodium-dodecyl sulfate, ammonium persulfate, TEMED, resazurin sodium salt, lithium lactate, iodonitrotetrazolium chloride, NAD, protease inhibitor cocktail, BSA, Bradford reagent, MPMS, Ac-DEVD-pNA, Ac-LEHD-pNa, Ac-YVAD-pNA, HEPES, Tween-20, NaCl, CHAPS, EDTA, DTT, INT, fluo-3-acetoxymethyl (Fluo-3AM), H33342, Pluronic F-127, GW9662 and TBC, were supplied by Sigma-Aldrich (USA). RNase was purchased from VWR (Poland), and PVDF membranes with a 0.45 µm pore size were acquired from Santa Cruz Biotechnology (USA). Antibodies targeting AhR were obtained from Proteintech (Germany), while those specific for PPARγ, mammalian target of rapamycin (mTOR), NF-κB, phospho-IκBα, IκBα, superoxide dismutases (SOD1, SOD2), catalase, nod like receptor protein 3 (NLRP3), binding immunoglobulin protein (Bip), the serine/threonine-protein kinase/endoribonuclease inositol-requiring enzyme 1 α (IRE1α), protein kinase R (PKR)-like endoplasmic reticulum kinase (PERK), activating transcription factor 6 (ATF6), Xbox binding protein (XBP1), eukaryotic initiation factor 2 alpha (eIF2α), activating transcription factor 4 (ATF4), nuclear factor erythroid 2-related factor 2 (Nrf2), Kelch-like ECH-associated protein 1 (Keap1), heme oxygenase (HO-1), NAD(P)H dehydrogenase (quinone) (NQO1), tumor necrosis factor-alpha (TNF-α), interleukin 1 beta (IL-1β), and IL-6 were sourced from ABClonal (USA) or Cell Signaling Technology (USA). Additionally, CAY10464 and honokiol were provided by Cayman Chemicals (USA).

### Cell Culture and Treatment

The HT-22 mouse hippocampal neuronal cell line (CRL-2260TM) was obtained from the American Type Culture Collection (ATTC, Manassas, VA, USA). Cells were cultured in Dulbecco’s Modified Eagle’s Medium (DMEM) without phenol red, supplemented with 10% fetal bovine serum (FBS), 100 U/mL penicillin and 0.10 mg/mL streptomycin. The cultures were maintained under standard conditions at 37 °C in a humidified atmosphere with 5% CO2 and passaged by trypsinization every 3 days when confluency reached approximately 80%. Cells were seeded in different culture plates depending on the specific assay: 96-well for resazurin reduction assay, LDH release assay, caspase-1, caspase-3, caspase-9 activity analyses, oxygen species production, and culture dishes for Western blot analysis. Cells were seeded at standard densities of 3.5 × 10^3^ cells/well or 1.6 × 10⁶ cells/dish and cultured for 24 h before treatment. For dose–response experiments, the medium was replaced with fresh medium containing varying concentrations of TBC (1, 10, 50, 100 nM and 1, 10, 50, 100 µM) for 48 h, unless otherwise indicated. Subsequently, cells were co-treated with 10 µM TBC (selected based on dose–response [37]) along with 1 µM CAY10464 (AhR antagonist), 1 µM GW9662 (PPARγ antagonist), or 15 µM honokiol (NF-κB inhibitor) for 24 and 48 h, with concentrations chosen based on prior studies [38–40].

### Resazurin Reduction Assay

The resazurin reduction assay was conducted according to the protocol described by Tabęcka-Łonczyńska et al. [37] without modifications. Briefly, cells were seeded into 96-well plates and exposed to 10 µM TBC, either alone or in combination with 1 µM CAY10464, 1 µM GW9662, or 15 µM honokiol, for 24 and 48 h at 37 °C. Following treatment, the medium was replaced with fresh medium containing 1% FBS and 10% resazurin (100 µL) and the cells were incubated for 60 min. Fluorescence was subsequently measured at excitation and emission wavelengths of 535 and 595 nm, respectively, using a FilterMax F5 Multi-Mode microplate reader (Molecular Devices, Corp., Sunnyvale, CA, USA).

### Lactate Dehydrogenase Release Assay

LDH release was assessed following the methodology described by Kaja et al. [41]. After 24 or 48 h of exposure to increasing concentrations of the test compound, 50 μL of the culture supernatant was transferred to a new 96-well plate. A reaction mixture was then added and the plate was incubated for 60 min in the dark. The remaining cells were frozen at − 80 °C for subsequent caspase-1, caspase-3, or caspase-9 activity assays. Absorbance was measured at 450 nm using a FilterMax F5 Multi-Mode microplate reader (Molecular Devices, Corp., Sunnyvale, CA, USA).

### Caspase-3, Caspase-9 and Caspase-1 Activities

Selected caspase activities were determined using the previously established protocol [42] without modifications. Following 24 or 48 h of exposure to 10 µM TBC, with or without co-treatment with 1 µM CAY10464, 1 µM GW9662, or 15 µM honokiol, cells were lysed in CAB buffer (50 mM HEPES, pH 7.4, 100 mM NaCl, 0.1% CHAPS, 1 mM EDTA, 10% glycerol and 10 mM DTT) at 4 °C for 10 min. Subsequently, specific caspase substrates (Ac-DEVD-pNA for caspase-3, Ac-LEHD-pNa for caspase-9 and Ac-YVAD-pNA for caspase-1) were added and absorbance was read at 405 nm after a 30-min incubation using a FilterMax F5 Multi-Mode microplate reader (Molecular Devices, Corp., Sunnyvale, CA, USA).

### Reactive Oxygen Species (ROS) Production

5 µM H2DCF-DA was used to evaluate the effect of the tested substances on the induction of ROS generation in HT-22 cells, as an important marker of the intracellular oxidative stress. The method was used as in Szychowski et al. (2016) [43]. Briefly, the cells were seeded in 96-well plates in DMEM medium supplemented with 10% FBS. After 24 h, the medium was replaced with a fresh one containing the fluorogenic dye at 5 μM concentration for 30 min. After this time, the dye was removed and the studied compounds were added to the plate. DCF fluorescence was measured after 6 and 24 h using a microplate reader (FilterMax F5) at maximum excitation and emission spectra of 485 and 535 nm, respectively. The data were analyzed using Multi-Mode Analysis software and were normalized to the fluorescence in the control group (% of control).

### Western Blotting

For Western blot analysis, HT-22 cells were seeded in culture dishes and cultured for 24 h. After exposure to 10 µM TBC, 1 µM GW9662, 1 µM CAY10464, or 15 µM honokiol, either individually or in combination, for 24 and 48 h, the cells were washed with PBS and lysed using ice-cold RIPA buffer supplemented with protease inhibitors. Protein concentration was determined using the BCA assay with BSA as the standard. An equal amount of proteins was separated by 7.5% SDS-PAGE and transferred onto PVDF membranes. Nonspecific binding was blocked with 1% BSA in TBST, followed by overnight incubation at 4 °C with primary antibodies. The membranes were washed and incubated for 1 h at room temperature with HRP-conjugated secondary antibodies. The specific details of antibodies and their concentrations are described in Table 1. Detection was performed using a chemiluminescent substrate (ECL) with the Western Blotting Luminol Reagent (Santa Cruz Biotechnology, Inc., Dallas, TX, USA) and visualized using a LiCor C DiGit imaging system. Densitometric analysis was conducted using GelQuantNET software, with band intensities normalized to GAPDH or *β*-actin.

**Table 1:** Specification of primary and secondary antibodies used in this study for Western blot method

Antibody	Antibody target	Host species	Dilution	Manufacturer	Cat #
**Primary**	Anti-AhR	Mouse	1:2000	Proteintech	67785–1-Ig
	Anti-PPARγ	Rabbit	1:1500	ABClonal	A11183
	Anti-mTOR	Rabbit	1:2000	ABClonal	A11355
	Anti-NF-κB	Mouse	1:3000	ABClonal	A10609
	Anti-Iκβα	Rabbit	1:1000	ABClonal	A19714
	Anti-phospho-IκBα	Rabbit	1:750	ABClonal	AP0707
	Anti-NLRP3	Rabbit	1:1000	ThermoFisher	PA5-79740
	Anti-SOD1	Rabbit	1:1000	CellSignaling	37385
	Anti-SOD2	Rabbit	1:2000	ABClonal	A19576
	Anti-catalase	Rabbit	1:1000	ABClonal	A11780
	Anti-Bip	Rabbit	1:1000	CellSignaling	3177
	Anti-IRE1α	Rabbit	1:1000	CellSignaling	3294
	Anti-PERK	Rabbit	1:1000	CellSignaling	3192
	Anti-ATF6	Rabbit	1:1000	CellSignaling	65880
	Anti-XBP-1 s	Rabbit	1:1000	CellSignaling	40435
	Anti-p-eIF2α	Rabbit	1:1000	CellSignaling	3398
	Anti-ATF4	Rabbit	1:1000	CellSignaling	11815
	Anti-Nrf2	Rabbit	1:1000	CellSignaling	12721
	Anti-Keap1	Rabbit	1:1000	CellSignaling	8047
	Anti-HO-1	Rabbit	1:1000	CellSignaling	43966
	Anti-NQO1	Rabbit	1:1000	CellSignaling	62262
	Anti-TNF-α	Rabbit	1:1000	CellSignaling	8184
	Anti-IL-1β	Rabbit	1:1000	CellSignaling	12703
	Anti-IL6	Rabbit	1:1000	CellSignaling	12153
	Anti-GAPDH	Mouse	1:100 000	ABClonal	AC033
	Anti-*β*-actin	Rabbit	1:1000	CellSignaling	4967
**HRP-conjugated antibodies**	Anti-Ms-HRP	Goat	1:50 000	ThermoFisher	31430
	Anti-Rb-HRP	Goat	1:50 000	ThermoFisher	31460

### Calcium Detection with Fluo-3AM

The fluorescence probe Fluo-3AM was employed to quantify intracellular calcium ion (Ca^2^⁺) levels, while Hoechst 33,342 (H33342) served as a nuclear counterstain. In brief, cells were treated with 10 µM TBC, 15 µM honokiol, or 1 µg/mL lipopolysaccharide (LPS) for 24 h. Following treatment, the cells were washed with phosphate-buffered saline (PBS) and incubated with Hoechst 33,342 and Fluo-3AM, both supplemented with 0.1% pluronic F-127 in a serum-free medium. The final concentrations of Hoechst 33,342 and Fluo-3AM were 10 µM and 5 µM, respectively. Next, the cells were incubated for 30 min at 37 °C in a 5% CO₂ atmosphere. After incubation, cells were washed with PBS and imaged using a fluorescence microscope (LSM 700, ZEISS). Fluorescence intensity was quantified using ImageJ software and expressed as corrected total cell fluorescence (CTCF), calculated according to the equation:

CTCF = Integrated Density − (Area of Selected Cell × Mean Fluorescence of Background).

Fluorescence measurements were performed on at least 10 images, with a minimum of 20 cells analyzed per image.

### Immunofluorescence NF-κB Staining

To confirm the effect of TBC on the translocation of NF-κB, HT-22 cells were seeded at a density of 2.5 × 10^4^ cells per well in 4-well cell culture slides and cultured for 24 h. Then, cells were treated with TBC (10 μM), LPS (1 μg/mL), or honokiol (15 μM) for 90 min. Then, cells were washed, fixed with paraformaldehyde (4%) for 20 min at room temperature and permeabilized with Triton X-100 (0.1%). Next, the cells were blocked with 3% BSA for 1 h at room temperature to suppress the nonspecific reaction after being washed with PBS four times. Afterward, they were incubated for another hour with anti-NF-κB primary antibody (1:100). After being washed with PBS three times, cells were incubated for another hour with a secondary antibody conjugated with Alexa Fluor 594 (1:100). Nuclei were counterstained with 0.3 μg/mL of the nuclear stain DAPI (Sigma Aldrich) for 5 min. Slides were permanently mounted using mounting medium (Ibidi GmbH, Martinsried, Germany). At last, the fluorescence signals of cells were caught with the fluorescence microscope (LSM 700, ZEISS). Fluorescence intensity was quantified using ImageJ software.

### Statistical Analysis

Data are presented as means ± standard deviation (SD) from three independent experiments (total replicates *n* = 9). Statistical analysis was performed using one-way analysis of variance (ANOVA), followed by Tukey’s post hoc test, with GraphPad Prism 8.0 software. Statistical significance was indicated as follows: ***p < 0.001, **p < 0.01 and *p < 0.05 compared to the control group; ###*p* < 0.001, ##*p* < 0.01 and #*p* < 0.05 compared to the TBC-exposed group.

## Results

### TBC Induces Time-Dependent Reduction in Metabolic Activity Without Affecting Membrane Integrity

**Fig. 1:**
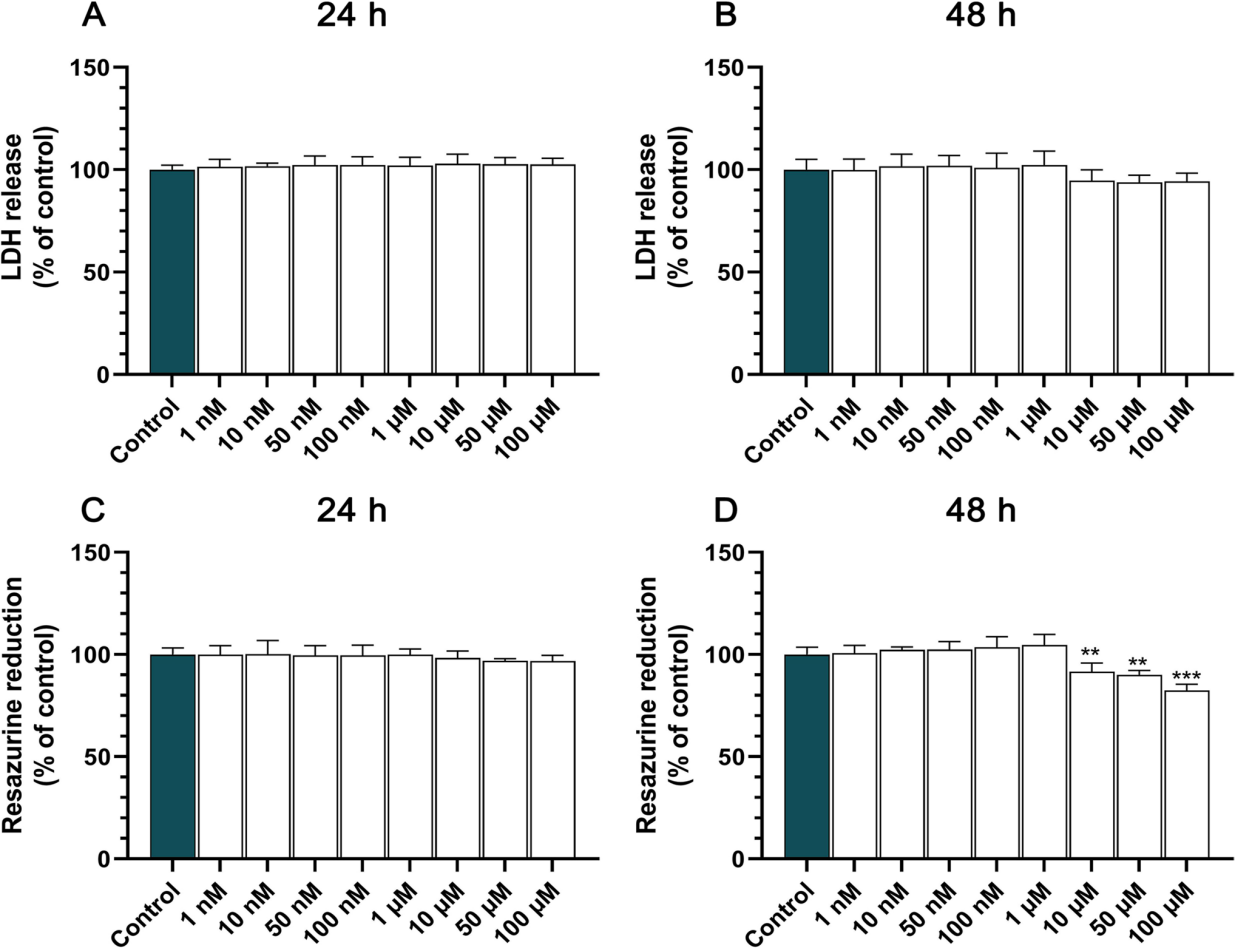
Effects of TBC on LDH release and metabolic activity in HT-22 cells. **A–B** LDH release was quantified after 24 h (**A**) and 48 h (**B**) of exposure to increasing concentrations of TBC (1 nM to 100 μM) to assess cellular membrane integrity. **C–D** Metabolic activity was evaluated using the resazurin reduction assay following 24 h (**C**) and 48 h (**D**) of treatment with TBC at the same concentrations. Data are presented as mean ± SD from three independent experiments, each consisting of six replicates per treatment group. Statistical significance is indicated as **p < 0.01 and ***p < 0.001 compared to control cells

In the beginning, the impact of TBC on membrane integrity in HT-22 cells was assessed by measuring lactate dehydrogenase (LDH) release. Our findings indicate that TBC, across the full range of tested concentrations (1–100 nM and 1–100 μM), did not induce significant LDH release after either 24 h (Fig. 1A) or 48 h (Fig. 1B) of exposure, suggesting no cytotoxic effect on cell membrane integrity under these conditions.

Subsequently, we evaluated the metabolic activity of HT-22 cells in response to TBC treatment using the resazurin reduction assay. After 24 h of exposure, no significant changes in metabolic activity were observed at any of the tested concentrations (Fig. 1C). However, after 48 h of exposure, a statistically significant reduction in metabolic activity was detected at higher concentrations of TBC. Specifically, a decrease of 8.43%, 9.95% and 17.55% was observed at 10, 50, and 100 µM concentrations, respectively, compared to the control group (Fig. 1D). These findings suggest that TBC exerts a time- and dose-dependent effect on cellular metabolism, with prolonged exposure leading to metabolic impairment.

### TBC Alters Caspase-1, −3 and −9 Activities in a Dose- and Time-Dependent Manner

**Fig. 2:**
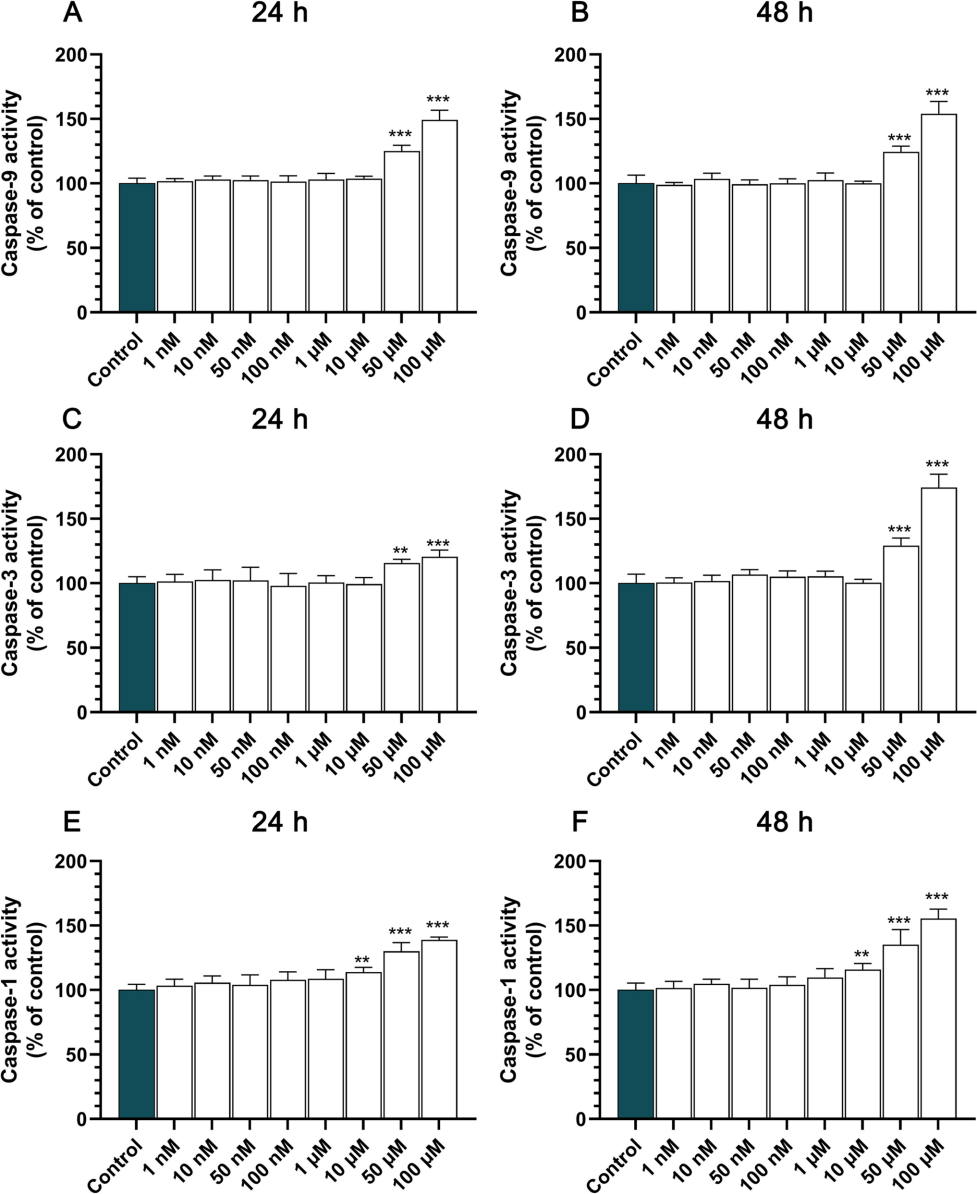
Impact of TBC on caspase activities in HT-22 cells. **A–B** Caspase-9 activity was measured after 24 h (**A**) and 48 h (**B**) of exposure to TBC at concentrations ranging from 1 nM to 100 μM. **C–D** Caspase-3 activity and **E–F** and caspases-1 were assessed at the same time points. Data are expressed as mean ± SD of three independent experiments, each including six replicates per treatment group. Statistical significance is denoted as **p < 0.01 and ***p < 0.001 relative to control cells

Next, we observed that a 24-h exposure to TBC in HT-22 cells led to a significant upregulation of caspase-9 activity at concentrations of 50 μM and 100 μM, with increases of 25.14% and 49.19% compared to the control (Fig. 2A). Following a 48-h exposure, caspase-9 activity exhibited further enhancement, with a 24.43% and 54.02% increase in cells treated with 50 μM and 100 μM of TBC, respectively (Fig. 2B). Caspase-3 activity demonstrated a similar pattern. After 24 h, cells treated with 50 μM and 100 μM of TBC showed a 15.71% and 20.66% increase in caspase-3 activity, respectively, compared to control (Fig. 2C). This effect was more pronounced after 48 h, with activity increases of 29.19% and 74.29% at 50 μM and 100 μM concentrations, respectively (Fig. 2D). Similarly, caspase-1 activity was elevated following TBC exposure. After 24 h, a significant increase in caspase-1 activity was observed at 10 μM, 50 μM and 100 μM, with elevations of 13.84%, 30.13% and 38.97%, respectively (Fig. 2E). At 48 h, the caspase-1 activity increased by 15.93%, 35.24% and 55.39% at the same concentrations, further confirming a dose- and time-dependent effect of TBC on apoptotic signaling pathways in neuronal cells (Fig. 2F).

### Treatment-Type-Dependent Cell Metabolic Activity Reduction and ROS Level Modulation

**Fig. 3:**
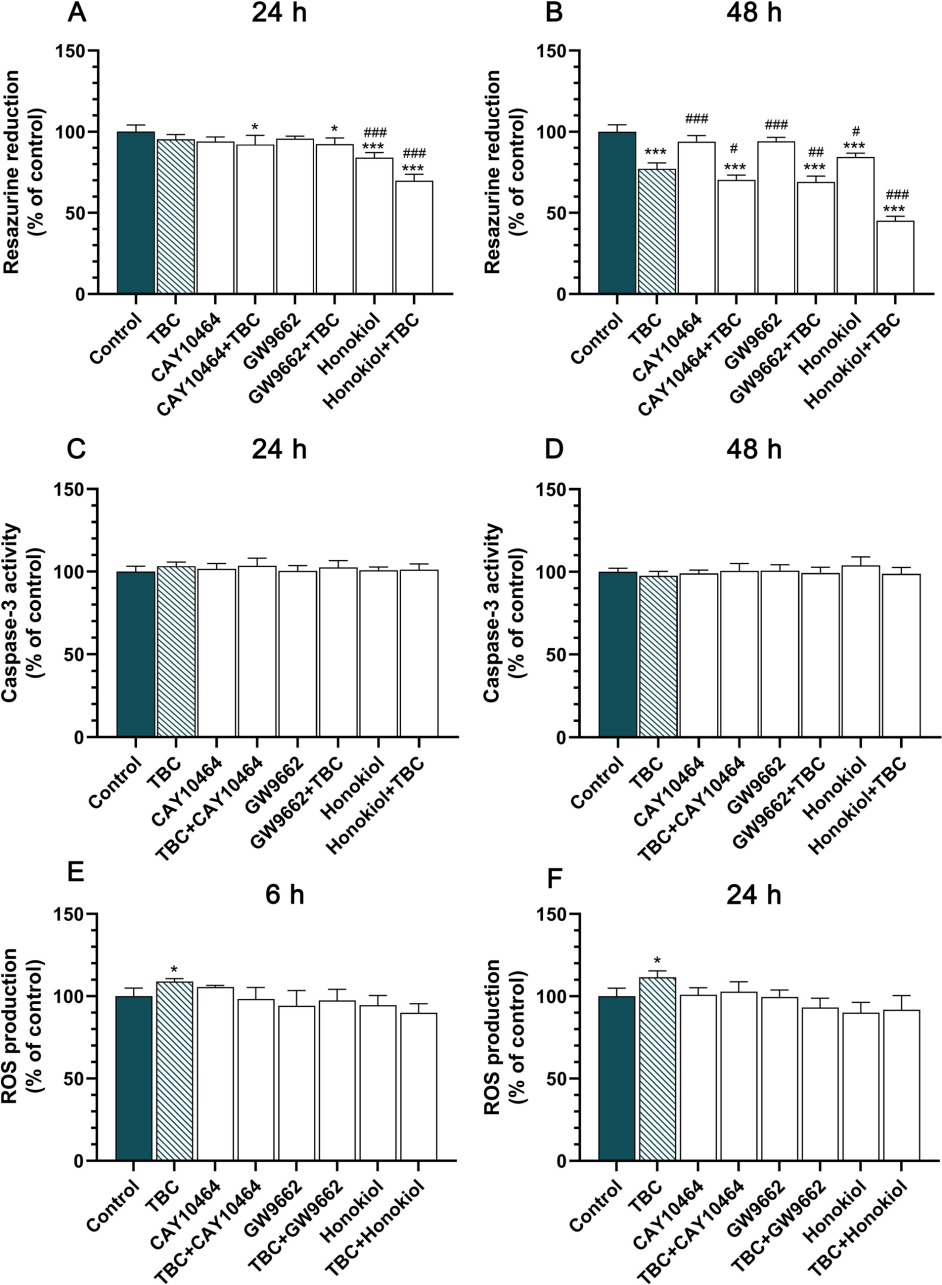
Effect of 10 μM TBC, 1 μM CAY10464, 1 μM GW9662 and 15 μM honokiol or cotreatment of TBC with CAY10464 or GW9662 or honokiol on resazurin reduction assay (**A, B**) and caspase-3 activity (**C, D**) and reactive oxygen species (**E, F**) on HT-22 cells exposed to the studied compounds for 24 and 48 h. Data are expressed as mean ± SD of three independent experiments, each of which comprised six replicates per treatment group. *p < 0.05, ***p < 0.001 vs. control cells; #*p* < 0.05, ##*p* < 0.01, ###*p* < 0.001 vs. cells treated by TBC alone. Reactive oxygen species (ROS) were quantified using H2DCFDA a cell-permeant indicator and expressed as a percentage relative to the control. Data are presented as mean ± SEM from three independent experiments. *p < 0.05 vs. control cells

To elucidate the involvement of specific signaling pathways in the action of TBC, we performed cotreatment experiments with selective antagonists and inhibitors. Cells were exposed to CAY10464 (an AhR antagonist), GW9662 (a PPARγ antagonist) and TBC for 24 h to assess the impact on metabolic activity, as measured by the resazurin reduction assay. No significant changes in metabolic activity were observed with CAY10464, GW9662, or TBC alone, as compared to control. However, co-treatment with CAY10464 and TBC or GW9662 and TBC led to a notable reduction in metabolic activity of 7.84% and 7.53%, respectively, compared to control. The impact was more pronounced with the NF-κB inhibitor honokiol, either alone or in combination with TBC, resulting in reductions of 15.96% and 30.18%, respectively. Additionally, significant differences in metabolic activity were observed between the honokiol and honokiol + TBC treatments compared to TBC alone (Fig. 3A). Upon extending the exposure to 48 h, TBC at 10 μM led to a 22.85% decrease in cell viability compared to control. Neither CAY10464 nor GW9662 at 1 μM concentrations significantly altered cell metabolism compared to the control. Nevertheless, the combination of CAY10464 with TBC and GW9662 with TBC resulted in decreases in metabolic activity of 29.70% and 30.90%, respectively. Honokiol alone and honokiol + TBC treatments showed reductions of 15.59% and 54.82%, respectively. The statistical analysis revealed significant differences for all treatment conditions relative to TBC alone, with notable differences observed for CAY10464, CAY10464 + TBC, GW9662, GW9662 + TBC, honokiol and honokiol + TBC (Fig. 3B). These results indicate that while AhR and PPARγ pathways minimally affect TBC-induced metabolic changes, NF-κB pathway modulation significantly influences TBC’s impact on cellular metabolic activity. Simultaneously, the assessment of caspase-3 activity revealed no significant alterations following treatment with all substances tested. Specifically, after 24 h and 48 h of exposure, caspase-3 activity remained statistically unchanged across all experimental conditions compared to control (Fig. 3C, D). Also, after both 6- and 24 h exposure of HT-22 cells to 10 µM TBC, we observed increased ROS production by 11.58% and 8.91%, respectively, compared to the control (Fig. 3E, F).

### TBC and Co-treatments Modulate AhR, PPARγ and IκBα Protein Expression in a Time- and Treatment-Dependent Manner

**Fig. 4:**
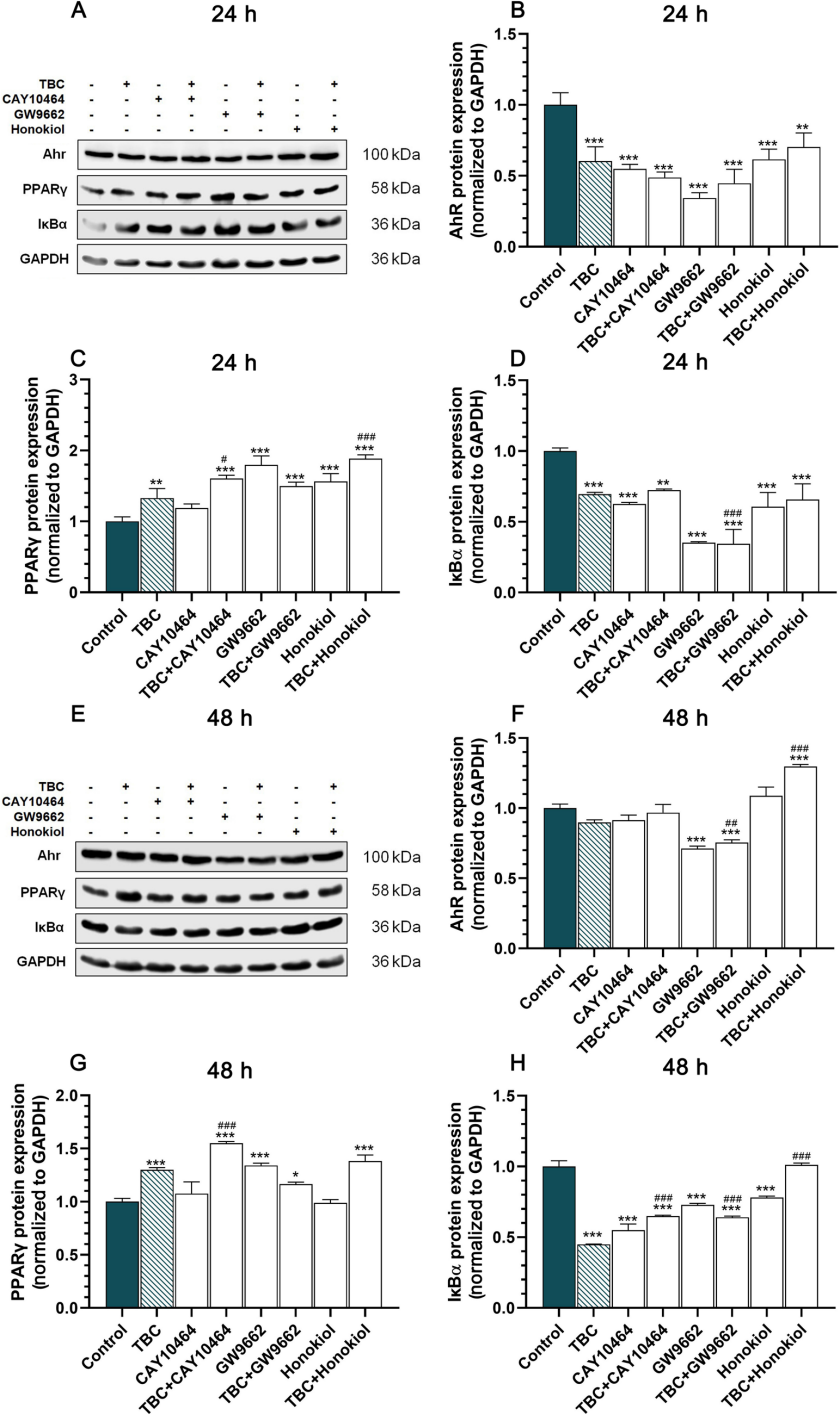
Western blot analysis of AhR, PPARγ and IκBα protein levels in HT-22 cells. **A, E** Representative Western blots depicting the expression of AhR (**B, F**), PPARγ (**C, G**) and IκBα (**D, H**) proteins in HT-22 cells treated with TBC (10 µM), CAY10464 (1 µM), GW9662 (1 µM), honokiol (15 µM) and their combinations for 24 and 48 h. Quantification of protein bands was performed using densitometry, with results expressed as a percentage relative to the control. Data are presented as mean ± SEM of three independent experiments. Each blot was stripped and reprobed with anti-GAPDH antibody for protein loading control. Statistical significance was assessed using Tukey’s test following one-way ANOVA. *p < 0.05, **p < 0.01 and ***p < 0.001 compared to the control group; #*p* < 0.05, ##*p* < 0.01 and ###*p* < 0.001 compared to TBC-treated group

Western blot analysis revealed a significant reduction in AhR protein expression following 24 h of exposure to TBC. Specifically, TBC treatment led to a 39.60% reduction in AhR expression compared to the control group. Similarly, CAY10464, an antagonist of AhR, also significantly decreased AhR protein expression by 45.19%. When cells were co-treated with CAY10464 and TBC, AhR expression was further reduced by 51.13%. In the group treated with GW9662, a PPARγ antagonist, a pronounced reduction of 65.64% was observed and the combination of GW9662 and TBC resulted in a 55.32% reduction. Honokiol treatment alone decreased AhR expression by 38.48%, while the combination of honokiol with TBC led to a 29.71% decrease. Importantly, no significant differences were noted between the TBC-treated groups when compared directly (Fig. 4A, B). After 24 h of TBC exposure, PPARγ protein expression was significantly upregulated by 32.79% compared to the control group. CAY10464 combined with TBC, GW9662 alone, GW9662 with TBC, honokiol alone and honokiol with TBC also significantly increased PPARγ expression by 60.47%, 79.73%, 49.86%, 56.26%, and 88.66%, respectively. In contrast, CAY10464 alone did not significantly alter PPARγ expression. Notably, significant upregulation was observed in CAY10464 + TBC and honokiol + TBC compared to TBC alone (Fig. 4A, C). In the case of IκBα, a consistent decrease in protein expression was observed across all treatment groups after 24 h of exposure. Specifically, TBC alone decreased IκBα expression by 30.44%, while CAY10464 alone led to a 37.49% decrease. The combination of CAY10464 + TBC resulted in a 27.62% reduction. GW9662 alone and in combination with TBC showed a pronounced decrease in IκBα expression by 64.77% and 65.54%, respectively. Similarly, honokiol alone and in combination with TBC decreased IκBα expression by 39.37% and 34.28%, respectively. A significant reduction was also noted in the GW9662 + TBC group compared to TBC alone (Fig. 3A, D).

After 48 h of incubation, fewer changes in AhR expression were observed. Neither TBC alone, CAY10464 alone, nor the combination of TBC with CAY10464, nor honokiol alone significantly affected AhR protein levels. However, GW9662 alone and GW9662 in combination with TBC resulted in a 28.82% and 24.45% decrease in AhR expression, respectively. In contrast, co-treatment with honokiol and TBC increased AhR expression by 29.75% compared to the control. Significant differences in AhR expression were also observed in GW9662 + TBC and honokiol + TBC compared to TBC alone (Fig. 4E, F).

Next, TBC treatment led to a 29.95% increase in PPARγ expression. Co-treatment with CAY10464 + TBC, GW9662 alone and honokiol + TBC also significantly elevated PPARγ expression by 55.05%, 34.01% and 38.27%, respectively. GW9662 combined with TBC resulted in a more moderate 16.56% increase. Interestingly, neither CAY10464 alone nor honokiol alone significantly altered PPARγ expression levels. However, co-treatment with CAY10464 + TBC resulted in a significant upregulation of PPARγ expression compared to TBC alone (Fig. 4E, G). Following 48 h of exposure, IκBα protein expression was further reduced in all experimental groups treated with TBC alone by 55.03%, CAY10464 alone by 45.11%, CAY10464 + TBC by 35.06%, GW9662 alone by 27.23%, GW9662 + TBC by 35.96% and honokiol alone by 21.89% compared to the control. Notably, the co-treatment of honokiol with TBC did not significantly alter IκBα protein levels. Additionally, significant changes in IκBα expression were observed in CAY10464 + TBC, GW9662 + TBC and honokiol + TBC when compared to TBC alone (Fig. 4E, H).

### TBC and Co-treatments Modulate mTOR/NF-κB/p-IκBα Pathways, NLRP3 Inflammasome and Pro-inflammatory Cytokines in a Time- and Treatment-Dependent Manner

**Fig. 5:**
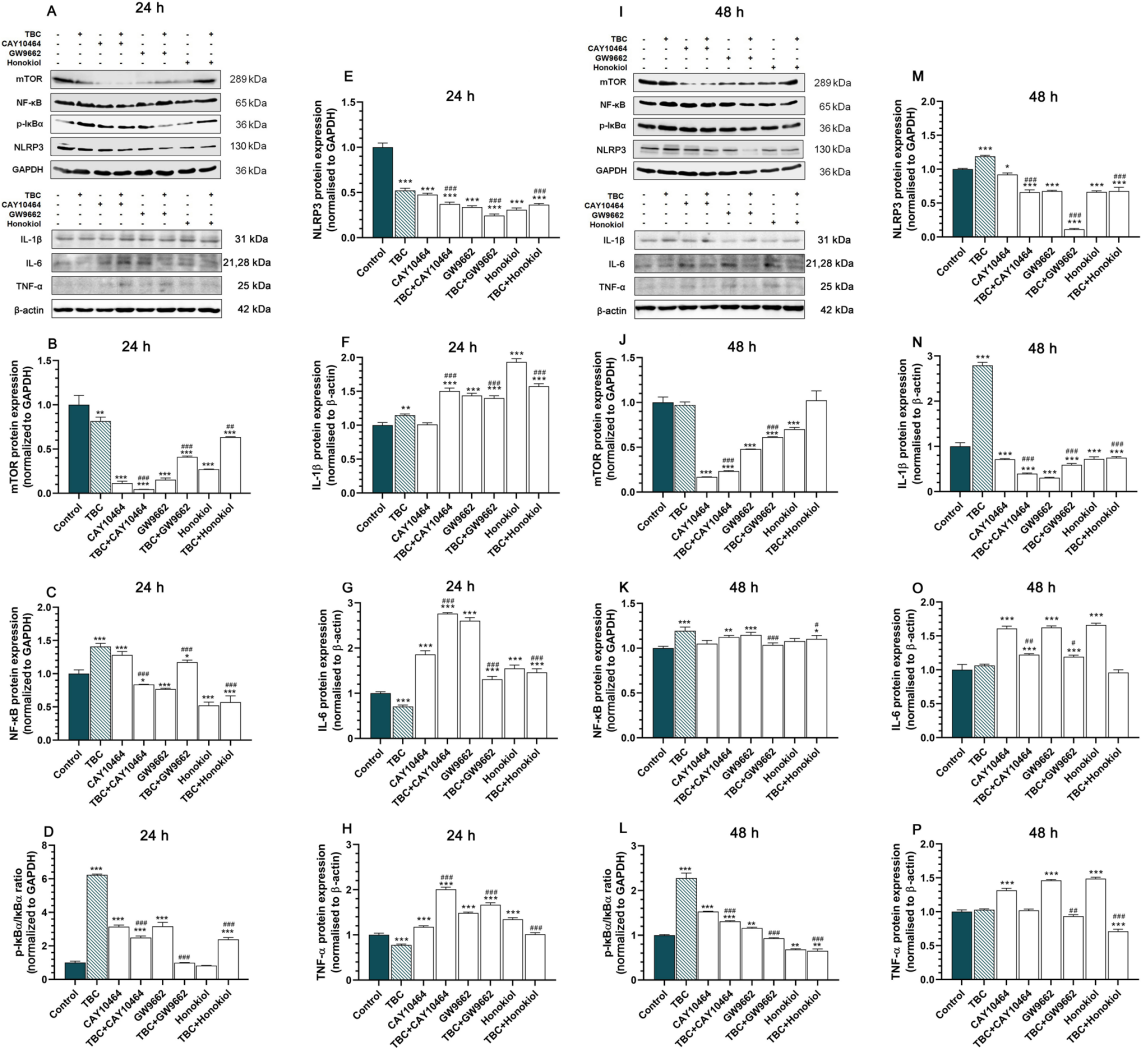
Western blot analysis of mTOR, NF-κB, p-IκBα, NLRP3, IL-1β, IL-6 and TNF-α protein levels in HT-22 cells. **A, I** Representative Western blots showing the expression levels of mTOR (**B, J**), NF-κB (**C, K**), p-IκBα (**D, L**), NLRP3 (**E, M**), IL-1β (**F, N**), IL-6 (**G, O**) and TNF-α (**H, P**) proteins in HT-22 cells treated with TBC (10 µM), CAY10464 (1 µM), GW9662 (1 µM), honokiol (15 µM) and their combinations for 24 and 48 h. Protein levels were quantified by densitometry and expressed as a percentage relative to the control. Data are presented as mean ± SEM from three independent experiments. The blots were stripped and reprobed with anti-GAPDH or β-actin antibodies for normalization of protein loading. Statistical significance was determined using Tukey’s test after one-way ANOVA. *p < 0.05, **p < 0.01 and ***p < 0.001 compared to the control group; #*p* < 0.05, ##*p* < 0.01 and ###*p* < 0.001 compared to TBC-treated group

In the HT-22 cells, following 24 h of exposure to TBC, we observed a significant decrease in mTOR protein expression across all experimental sets. Specifically, TBC alone reduced mTOR expression by 18.46% compared to the control. More pronounced reductions were observed in the groups treated with CAY10464 alone (88.55%), co-treated with CAY10464 and TBC (95.49%), GW9662 alone (84.63%) and GW9662 combined with TBC (58.99%). Additionally, honokiol alone and in combination with TBC decreased mTOR expression by 72.75% and 36.58%, respectively. Furthermore, when compared to TBC alone, there were significant further reductions in mTOR expression in the co-treatment groups for CAY10464 + TBC or GW9662 + TBC and honokiol + TBC (Fig. 5A, B). A similar trend was observed after 48 h of exposure, with some exceptions. Specifically, mTOR protein expression decreased in the CAY10464-treated by 83.05%, CAY10464 + TBC co-treated by 76.69%, GW9662-treated by 52.01% and GW9662 + TBC by 38.68%, as well as in the honokiol-treated group by 29.85% compared to the control. However, TBC alone and honokiol + TBC did not significantly affect mTOR expression after 48 h. Notable downregulation was observed in the CAY10464 + TBC and GW9662 + TBC groups compared to TBC alone (Fig. 5I, J).

Similarly, after 24 h of exposure to 10 µM TBC, NF-κB protein expression increased by 40.84% compared to the control. Increased NF-κB expression was also noted in the CAY10464-treated group by 28.12% and the GW9662 + TBC co-treatment group by 17.19%. In contrast, co-treatment with CAY10464 and TBC led to a 23.01% decrease in NF-κB expression and significant reductions were also seen in the GW9662 alone by 16.23%, honokiol alone by 47.69%, and honokiol + TBC by 42.90% groups compared to control. Additionally, when compared to TBC treatment alone, we observed a significant reduction in NF-κB protein expression in the groups co-treated with CAY10464 and TBC, GW9662 and TBC and honokiol and TBC (Fig. 5A, C). Additionally, 48 h of TBC exposure resulted in a 19.26% increase in NF-κB protein expression compared to the control. Increases were also noted in the CAY10464 + TBC by 12.30%, GW9662-treated by 14.85% and honokiol + TBC co-treated groups by 10.34% compared to the control. However, significant downregulation of NF-κB was observed in the GW9662 + TBC and honokiol + TBC groups when compared to TBC alone (Fig. 5I, K).

Regarding p-IκBα protein expression, a 24-h exposure to 10 µM TBC alone resulted in a substantial 522.90% increase compared to the control. Similar increases were observed in the CAY10464-treated by 214.20%, CAY10464 + TBC co-treated by 149.43% and GW9662-treated by 216.68% groups. Co-treatment with honokiol and TBC also led to a 138.37% increase. Notably, in comparison to TBC alone, significant decreases in p-IκBα expression were observed in the CAY10464 + TBC, GW9662 + TBC and honokiol + TBC groups (Fig. 5A, D). Finally, after 48 h of TBC exposure, p-IκBα expression increased by 127.72% compared to the control. CAY10464 alone and CAY10464 + TBC co-treatment increased p-IκBα expression by 52.79% and 31.13%, respectively. GW9662 alone also increased p-IκBα expression by 16.24%. In contrast, honokiol alone and honokiol + TBC treatment decreased p-IκBα expression by 31.60% and 34.90%, respectively, compared to the control. Significant decreases in p-IκBα expression were also observed in the CAY10464 + TBC, GW9662 + TBC and honokiol + TBC groups compared to TBC alone (Fig. 5I, L).

At 24 h, NLRP3 expression was markedly reduced in all groups compared to control for TBC (48.85%), CAY10464 (52.50%), CAY10464 + TBC (62.83%), GW9662 (66.20%), GW9662 + TBC (75.51%), honokiol (69.22%) and honokiol + TBC (63.30%) (Fig. 5A, E). Similar reductions persisted at 48 h in all groups except for TBC, which showed an 18% increase. Notably, CAY10464 + TBC, GW9662 + TBC and honokiol + TBC groups demonstrated further reductions compared to TBC alone. The remaining groups showed reduced NLRP3 expression levels for CAY10464 by 17.02%, CAY10464 by 34.13%, GW9662 by 32.42%, TBC with GW9662 by 88.73%, honokiol by 33.59% and TBC with honokiol by 32.39%. Furthermore, NLRP3 expression was significantly lower in the CAY10464 + TBC, GW9662 + TBC and honokiol + TBC groups when compared to TBC alone (Fig. 5I, M).

After 24 h of exposure to TBC, the level of IL-1β was upregulated for TBC by 14.78%, CAY10464 + TBC by 50.37%, GW9662 by 43.79%, GW9662 + TBC by 39.95%, honokiol by 93.32% and honokiol + TBC by 57.35% compared to control. Increased level of IL-1β was observed in the CAY10464 + TBC, the GW9662 + TBC and honokiol + TBC groups compared to TBC alone (Fig. 5A, F). After 48 h, we observed increased IL-1β expression in TBC by 179.08%, but all other groups were reduced compared to control: CAY10464 by 28.73%, CAY10464 + TBC by 60.30%, GW9662 by 69.43%, GW9662 + TBC by 40.79%, honokiol by 27.84 and honokiol + TBC by 25.02%. Also, IL-1β expression was reduced in the CAY10464 + TBC, GW9662 + TBC and honokiol + TBC groups compared to TBC alone (Fig. 5I, N).

For IL-6, we observed a decrease in the TBC group by 29.30%. On the contrary, for the other groups, there was an increase for CAY10464 by 85.59%, CAY10464 + TBC by 176.08%, GW9662 by 160.45%, GW9662 + TBC by 30.80%, honokiol by 54.78% and honokiol + TBS 46.07% compared to the control. However, in comparison to the TBC group, we noted a significant increase in IL-6 expression for CAY10464 + TBC, GW9662 + TBC and honokiol + TBC (Fig. 5A, G). In turn, no significant changes in IL-6 expression were observed in the TBC group after 48 h. However, significant upregulation was noted in the CAY10464 (60.86%), CAY10464 + TBC (22.30%), GW9662 (62.49%), GW9662 + TBC (19.26%) and honokiol (66.13%) groups compared to control. Compared to TBC alone, increased IL-6 expression was observed in CAY10464 + TBC and GW9662 + TBC (Fig. 5I, O).

TBC exposure decreased TNF-α expression by 22.33% after 24 h, whereas other treatments, including CAY10464 (17.51%), CAY10464 + TBC (100.95%), GW9662 (48.48%), GW9662 + TBC (66.66%) and honokiol (34.43%), showed significant increases compared to control (Fig. 5A, H). At 48 h, TNF-α levels were upregulated in CAY10464 (31.58%), GW9662 (46.27%) and honokiol (48.83%) but were significantly reduced in honokiol + TBC (28.89%). Both GW9662 + TBC and honokiol + TBC groups exhibited lower TNF-α expression compared to TBC alone (Fig. 5I, P).

### Impact of TBC and Co-treatments on Inflammatory Markers and ER Stress Pathways in HT-22 Cells

**Fig. 6:**
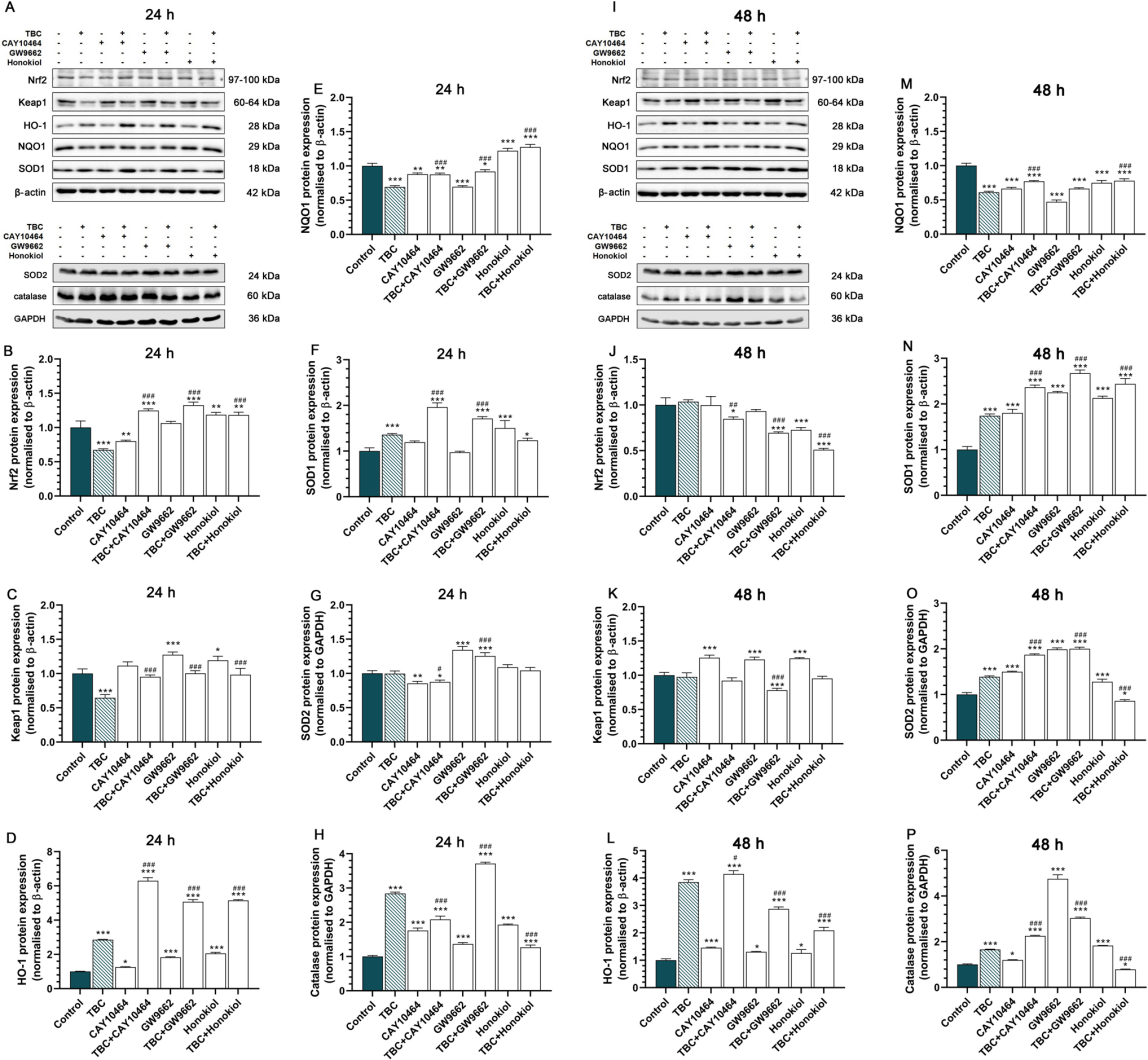
Western blot analysis of Nfr2, Keap1, HO-1, NQO1, SOD1, SOD2 and catalase protein levels in HT-22 cells. **A, J** Representative Western blots showing the expression levels of Nrf2 (**B, J**), Keap1 (**C, K**), HO-1 (**D, L**), NQO1 (**E, M**), SOD1 (**F, N**), SOD2 (**G, O**) and catalase (**H, P**) proteins in HT-22 cells treated with TBC (10 µM), CAY10464 (1 µM), GW9662 (1 µM), honokiol (15 µM) and their combinations for 24 and 48 h. The blots were stripped and reprobed with anti-*β*-actin and anti-GAPDH antibodies for normalization of protein loading. Statistical significance was determined using Tukey’s test after one-way ANOVA. *p < 0.05, **p < 0.01 and ***p < 0.001 compared to the control group; #*p* < 0.05, ##*p* < 0.01 and ###*p* < 0.001 compared to TBC-treated group

After 24 h, Nrf2 expression was significantly reduced in TBC by 32.71% and CAY10464 by 19.92% compared to the control. However, Nrf2 levels were increased in CAY10464 + TBC by 24.73%, GW9662 + TBC by 32.55%, honokiol by 18.52% and honokiol + TBC by 18.28%. Compared to TBC alone, Nrf2 expression was elevated in all co-treatment groups (Fig. 6A, B). At 48 h, Nrf2 expression was significantly reduced in CAY10464 + TBC by 15.31%, GW9662 + TBC by 30.56%, honokiol by 27.37% and honokiol + TBC by 49.07% compared to the control. Relative to TBC alone, Nrf2 expression was significantly decreased in the CAY10464 + TBC group and in GW9662 + TBC and honokiol + TBC groups (Fig. 6I, J).

Analysis of Keap1 expression revealed a significant reduction in TBC by 35.50%, in contrast to GW9662 by 27.34% and honokiol by 19.21%, we observed an increase compared to the control group. Moreover, in co-treatment groups with TBC, we observed a significant increase compared to TBC alone (Fig. 6A, C). However, after 48 h exposure to TBC, the level of Keap1 was upregulated in CAY10464 by 25.57%, GW9662 by 22.96% and honokiol by 24.54% compared to control. Only in GW9662 + TBC the Keap1 expression was downregulated by 21.97% compared to control. Similarly, in GW9662 + TBC, a reduced level of Keap1 was observed after TBC related to TBC alone (Fig. 6I, K).

HO-1 levels were significantly elevated across all groups after 24 h for TBC (185.47%), CAY10464 (25.85%), CAY10464 + TBC (529.80%), GW9662 (82.82%), GW9662 + TBC (46.97%), honokiol (105.88%) and honokiol + TBC (415.33%) (Fig. 6A, D). Further increases were observed at 48 h, with prominent elevations in TBC (284.83%), CAY10464 (45.50%), CAY10464 + TBC (314.84%), GW9662 (30.43%), GW9662 + TBC (187.31%) and honokiol (108.96%). However, reductions were noted in GW9662 + TBC and honokiol + TBC groups compared to TBC alone (Fig. 6I, L).

Following 24 h of TBC exposure, NQO1 expression was significantly reduced in the TBC by 30.80%, CAY10464 by 12.04%, CAY10464 + TBC by 12.43%, GW9662 by 30.37% and GW9662 + TBC by 8.20% groups. Conversely, NQO1 levels were elevated in the honokiol by 22.07% and honokiol + TBC by 27.63% groups compared to the control. Compared to TBC alone, upregulated NQO1 expression was observed in the CAY10464 + TBC, GW9662 + TBC and honokiol + TBC groups (Fig. 6A, E). After 48 h, NQO1 levels were significantly downregulated across all groups relative to control: TBC by 38.90%, CAY10464 by 33.63%, CAY10464 + TBC by 22.97%, GW9662 by 52.84%, GW9662 + TBC by 33.66%, honokiol by 25.01% and honokiol + TBC by 22.12%. However, NQO1 expression in the CAY10464 + TBC and honokiol + TBC groups was increased compared to TBC alone (Fig. 6I, M).

After 24 h of TBC exposure, SOD1 protein expression was significantly elevated in the following groups compared to control: TBC alone (35.91%), CAY10464 + TBC (96.17%), GW9662 + TBC (71.13%), honokiol (50.28%) (***, *p* < 0.001, each) and honokiol + TBC (23.45%). Notably, CAY10464 + TBC and GW9662 + TBC groups exhibited significantly higher SOD1 levels compared to TBC alone (Fig. 6A, F). After 48 h, further increases in SOD1 were observed across all groups, including TBC (74.14%), CAY10464 (80.35%), CAY10464 + TBC (136.54%), GW9662 (125.00%), GW9662 + TBC (167.49%), honokiol (112.88%) and honokiol + TBC (143.96%). CAY10464 + TBC, GW9662 + TBC and honokiol + TBC groups maintained significantly elevated levels relative to TBC alone (Fig. 6I, N).

SOD2 expression analysis revealed a 14.62% reduction in the CAY10464 group and a 12.39% reduction in the CAY10464 + TBC group after 24 h compared to the control. In contrast, GW9662 and GW9662 + TBC treatments increased SOD2 expression by 34.18% and 25.49%, respectively (Fig. 6A, G). At 48 h, SOD2 expression increased in most groups: TBC (38.80%), CAY10464 (49.84%), CAY10464 + TBC (87.22%), GW9662 (98.86%), GW9662 + TBC (100.10%) and honokiol (27.98%) except for honokiol + TBC, which showed a 14.21% reduction. Significant increases were noted in CAY10464 + TBC and GW9662 + TBC groups, with a reduction in honokiol + TBC compared to TBC alone (Fig. 6I, O).

In turn, catalase expression was consistently elevated after 24 h across all groups: TBC (183.73%), CAY10464 (75.81%), CAY10464 + TBC (108.29%), GW9662 (36.54%), GW9662 + TBC (271.29%), honokiol (92.81%) and honokiol + TBC (26.94%). However, the CAY10464 + TBC and honokiol + TBC groups showed reduced expression levels, while GW9662 + TBC had significantly higher levels compared to TBC alone (Fig. 6A, H). After 48 h, catalase levels were upregulated in most groups, including TBC (65.99%), CAY10464 (19.57%), CAY10464 + TBC (125.37%), GW9662 (375.24%; ***, *p* < 0.001), GW9662 (375.24%) and GW9662 + TBC (203.18%). In contrast, honokiol + TBC reduced catalase expression by 21.45% compared to control. Relative to TBC alone, CAY10464 + TBC and GW9662 + TBC groups showed increased expression, while honokiol + TBC exhibited reduced levels (Fig. 6I, P).

### Regulation of Endoplasmic Reticulum Stress Pathways by TBC and Combined Treatments in HT-22 Cells

**Fig. 7:**
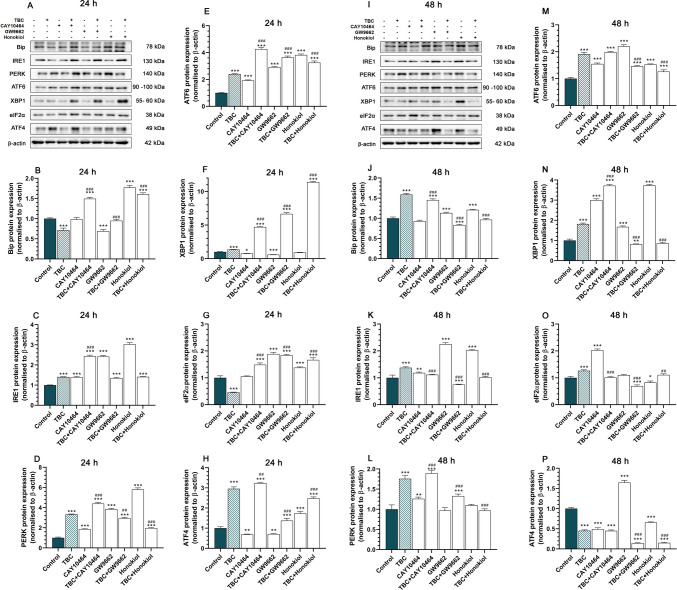
Western blot analysis of Bip, IRE1, PERK, ATF6, XBP1, eIF2α and ATF4 protein levels in HT-22 cells. **A, I** Representative Western blots showing the expression levels of Bip (**B, J**), IRE1 (**C, K**), PERK (**D, L**), ATF6 (**E, M**), XBP1 (**F, N**) eIF2α (**G, O**) and ATF4 (**H, P**) proteins in HT-22 cells treated with TBC (10 µM), CAY10464 (1 µM), GW9662 (1 µM), honokiol (15 µM) and their combinations for 24 and 48 h. Protein levels were quantified by densitometry and expressed as a percentage relative to the control. Data are presented as mean ± SEM from three independent experiments. The blots were stripped and reprobed with anti-β-actin antibody for normalization of protein loading. Statistical significance was determined using Tukey’s test after one-way ANOVA. Anti-*β*-actin, **p < 0.01 and ***p < 0.001 compared to the control group; #*p* < 0.05, ##*p* < 0.01 and ###*p* < 0.001 compared to TBC-treated group

At 24 h, TBC exposure led to a significant reduction in Bip expression by 28.48%, as well as GW9662 by 30.97% compared to control. Conversely, increased Bip levels were observed in the CAY10464 + TBC by 49.44%, honokiol by 77.21% and honokiol + TBC by 60.75%. Significant increases were also noted in the CAY10464 + TBC, W9662 + TBC and honokiol + TBC groups compared to TBC alone (Fig. 7A, B). After 48 h, Bip expression was upregulated in the TBC by 58.98%, CAY10464 + TBC by 45.44%, GW9662 by 13.17% and honokiol by 21.02% groups compared to the control. However, downregulation was observed in the GW9662 + TBC group by 16.56%. Compared to TBC alone, significant reductions in Bip levels were found in the CAY10464 + TBC, GW9662 + TBC, and honokiol + TBC groups (Fig. 7I, J).

At 24 h post-TBC exposure, IRE1 expression was significantly upregulated in TBC (40.37%), CAY10464 (39.23%), CAY10464 + TBC (143.16%), GW9662 (141.53%), GW9662 + TBC (35.10%), honokiol (202.65%) and honokiol + TBC (41.18%) compared to the control group. When compared to TBC alone, IRE1 levels were significantly elevated only in the CAY10464 + TBC group (Fig. 7A, C). At 48 h, increased IRE1 expression was observed in TBC by 37.50%, CAY10464 by 18.43%, GW9662 by 125.37% and honokiol by 102.60%, while a decrease was noted in GW9662 + TBC by 25.02% compared to the control. Relative to TBC alone, IRE1 expression significantly decreased in all co-treatment groups (Fig. 7I, K).

PERK expression was significantly upregulated after 24 h of exposure in TBC (232.69%), CAY10464 (84.89%), CAY10464 + TBC (339.37%), GW9662 (284.24%), GW9662 + TBC (194.13%), honokiol (479.83%) and honokiol + TBC (97.08%). Relative to TBC alone, PERK levels were elevated in the CAY10464 + TBC group but reduced in GW9662 + TBC and honokiol + TBC (Fig. 7A, D). At 48 h, PERK expression remained elevated in TBC by 75.74%, CAY10464 by 26.10%, CAY10464 + TBC by 89.30% and GW9662 + TBC by 32.96% compared to control. Relative to TBC alone, CAY10464 + TBC exhibited increased PERK expression, while GW9662 + TBC and honokiol + TBC demonstrated decreased expression (Fig. 7I, L).

At 24 h, ATF6 expression was significantly upregulated in TBC (140.15%), CAY10464 (93.28%), CAY10464 + TBC (326.02%), GW9662 (190.82%), GW9662 + TBC (263.74%), honokiol (282.16%) and honokiol + TBC (225.10%). Furthermore, ATF6 levels were significantly increased in all TBC co-treatment groups relative to TBC alone (Fig. 7A, E). Similarly, after 48 h, ATF6 expression remained elevated in TBC (91.07%), CAY10464 (54.51%), CAY10464 + TBC (98.26%), GW9662 (120.07%), GW9662 + TBC (46.44%), honokiol (52.89%) and honokiol + TBC (26.14%). Decreased ATF6 expression was observed in GW9662 + TBC and honokiol + TBC groups compared to TBC alone (Fig. 7I, M).

After 24 h, XBP1 expression was significantly upregulated in the TBC (35.75%), CAY10464 + TBC (371.71%), GW9662 + TBC (566.93%) and honokiol + TBC (1040.76%) groups compared to the control. Conversely, XBP1 was downregulated in CAY10464 by 21.61% and GW9662 by 40.96%. Compared to TBC alone, XBP1 levels were significantly higher in all co-treatment groups (Fig. 7A, F). At 48 h, XBP1 expression remained elevated in TBC (81.86%), CAY10464 (199.95%), CAY10464 + TBC (271.87%), GW9662 (67.49%) and honokiol (272.85%) groups compared to the control. However, a reduction was observed in GW9662 + TBC by 18.17%. Relative to TBC alone, XBP1 expression was elevated in CAY10464 + TBC but decreased in GW9662 + TBC and honokiol + TBC groups (Fig. 7I, N).

At 24 h, eIF2α expression was significantly decreased in the TBC group by 54.43% compared to control. Conversely, increased levels were observed in the CAY10464 + TBC (48.93%), GW9662 (88.67%), GW9662 + TBC (83.57%), honokiol (37.78%) and honokiol + TBC (66.57%) groups. Compared to TBC alone, eIF2α levels were significantly elevated in the CAY10464 + TBC, GW9662 + TBC and honokiol + TBC groups (Fig. 7A, G). After 48 h, eIF2α levels increased in TBC (26.35%) and CAY10464 (102.74%), while they decreased in GW9662 + TBC by 30.52% and honokiol by 17.26% compared to control. Relative to TBC alone, significant reductions were observed in the CAY10464 + TBC, GW9662 + TBC and honokiol + TBC groups (Fig. 7I, O).

In HT-22 cells exposed for 24 h, ATF4 expression increased significantly in TBC (195.45%), CAY10464 + TBC (223.65%), GW9662 + TBC (37.41%), honokiol (73.20%) and honokiol + TBC (147.65%) groups compared to control. However, decreased levels were detected in the CAY10464 by 30.54% and GW9662 by 28.70% groups (Fig. 7A, H). After 48 h, ATF4 expression was upregulated in the GW9662 group by 64.58% but significantly downregulated in the TBC (54.77%), CAY10464 (50.75%), CAY10464 + TBC (55.11%), GW9662 + TBC (85.55%), honokiol (34.67%) and honokiol + TBC (84.92%) groups compared to the control. Relative to TBC alone, ATF4 expression was significantly lower in the GW9662 + TBC and honokiol + TBC groups (Fig. 7I, P).

### TBC Induces Calcium Release in HT-22 Cells

**Fig. 8:**
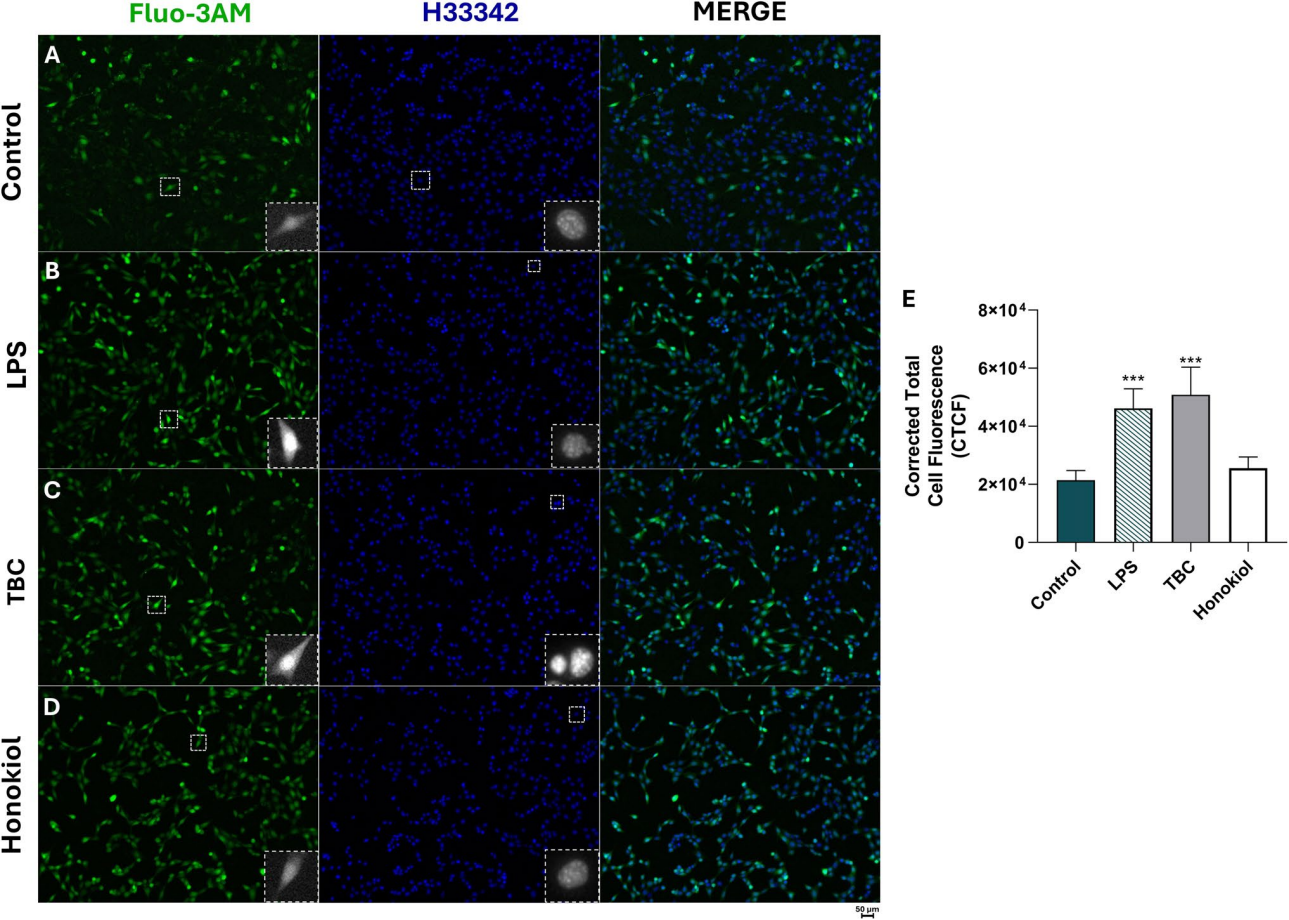
Confocal imaging with Hoechst 33,342 (H33342) and Fluo–3AM staining of the HT-22 cells after exposure to 1 µg/mL of LPS (**B**), 10 µM of TBC (**C**), 15 µM of honokiol (**D**) and control group (**A**) after 24 h treatment. LPS was used as positive control. The 100 × magnification was used. Dashed squares indicate the regions used for the zoom. Corrected total cell fluorescence intensity (**E**). Data are expressed as a mean with standard deviation. Statistically significant values were determined by Tukey’s test for each study group. ***p < 0.001, compared to the control cells

Confocal fluorescence microscopy, along with corrected total cell fluorescence (CTCF) analysis, was employed to investigate the direct effects of TBC on intracellular Ca^2^⁺ concentration in HT-22 cells. Fluorescent staining using Fluo-3AM revealed a significant increase in intracellular calcium pool following lipopolysaccharide (LPS) treatment by 2.74 × 10^4^ RFU compared to control (Fig. 8B, E). Similarly, TBC treatment resulted in elevated intracellular calcium levels by 2.94 × 10^4^ RFU compared to the control group (Fig. 8C, E). In contrast, no significant change in RFU was observed in the honokiol-treated group after 24 h (Fig. 8D, E). Furthermore, no noticeable alterations in nuclear morphology were detected in any of the treatment groups.

### NF-κB p65 Nuclear Translocation in HT-22 Cells Induced by LPS, TBC and Honokiol Treatments

**Fig. 9:**
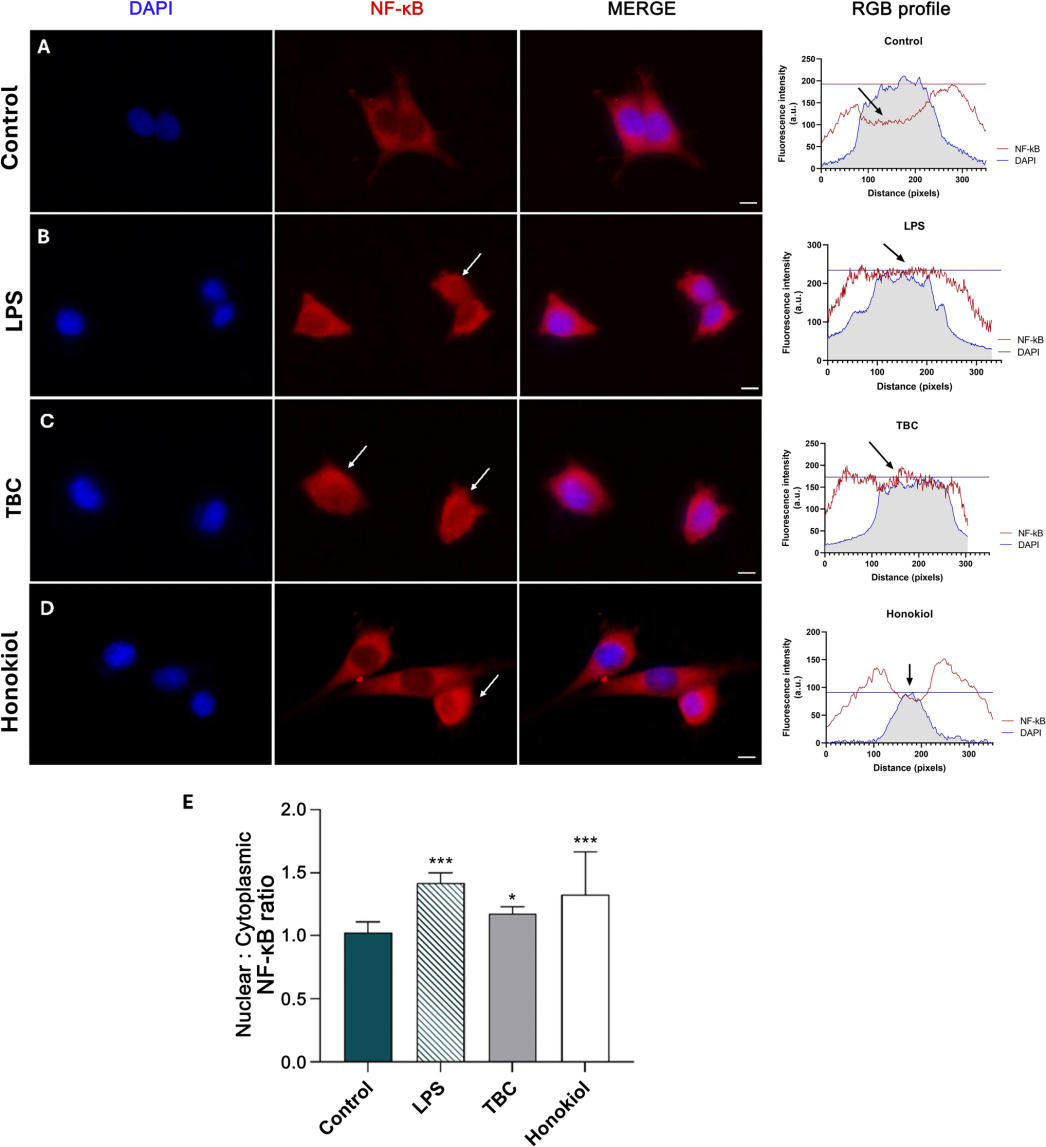
Confocal immunofluorescence staining of NF-κB and quantification of nuclear:cytoplasmic ratio of NF-κB staining in unstimulated HT-22 cells (**A**) and after 90 h stimulation with 10 μM TBC (**B**), 1 µg/mL LPS (**C**), or 15 μM honokiol (**D**). The 1000 × magnification was used. Quantitative analysis was performed by the ImageJ software on a minimum of 50 cells for each sample. Data are expressed as a mean with standard deviation. Statistically significant values were determined by Tukey’s test for each study group. *p < 0.05 and ***p < 0.001, compared to the control cells. The intensity of DAPI and Alexa Fluor 594 was plotted using ImageJ software for quantification of fluorescence intensity. The intensity (in arbitrary unit) was taken along the *Y*-axis and distance (in pixels) was plotted along the *X*-axis. For control, TBC, LPS and honokiol groups, the red line indicates the intensity of Alexa Fluor 594 and the blue line indicates the intensity of DAPI. Scale bar indicates 10 μm

To directly observe the translocation of NF-κB p65 in HT-22 cells, immunofluorescence staining was performed and the images are shown in Fig. 9. LPS stimulation induced a significant translocation of NF-κB p65 from the cytoplasm to the nucleus, compared to the unstimulated control (Fig. 9A, B, E). TBC-treated cells showed an elevated localization of NF-κB inside the nucleus (Fig. 9C, E). Honokiol treatment also resulted in increased levels of NF-κB p65 inside the nuclei (Fig. 9D, E).

## Discussion

The ubiquitous presence of tris(2,3-dibromopropyl) isocyanurate (TBC) in the environment and its adverse effects on various tissues are well-documented [18, 44]. Our study investigates the impact of TBC on hippocampal cells by evaluating cytotoxicity, apoptotic markers and involvement of key molecular pathways.

Contrasting with our findings, previous studies have reported differential cytotoxic responses to TBC exposure. For instance, TBC did not affect LDH release in undifferentiated and differentiated human neuroblastoma SH-SY5Y cells following 7 days of exposure. However, significant LDH release was observed after 24 h of exposure in differentiated SH-SY5Y cells (14-day differentiation) across a broad range of concentrations (1–100 nM and 1–100 µM) [21]. Similarly, A549 cells exhibited increased LDH release at higher TBC concentrations (10–100 µM) [45]. In contrast, our resazurin reduction assay revealed a decrease in cellular metabolism only after 48 h of exposure to TBC at 10, 50 and 100 µM. These results suggest that while TBC may not be overtly toxic to hippocampal cells, it likely impairs cellular proliferation after prolonged exposure. This finding is consistent with similar results obtained in astrocyte cultures, where TBC exposure (10–100 µM) reduced cell numbers, likely due to inhibited cell proliferation [46]. Other studies also reported decreased metabolic activity in SH-SY5Y neuroblastoma cells after TBC exposure (12.5–100 µM) [16] and cytotoxicity in primary rat neurons after exposure to 10–50 µM TBC for 48 h [18]. Zebrafish embryos (*Danio rerio*) also exhibited cytotoxicity at 1 µg/mL TBC after 72 h [47]. However, no toxicity was observed in studies on A549 cells [45] or human hepatocarcinoma (Hep-G2) cells [15, 48]. In Hep-G2 cells, high cytochrome P450 activity, which is crucial for xenobiotic metabolism, might offer protection against TBC-induced toxicity [48]. While our findings indicate that TBC does not cause immediate cytotoxicity in HT-22 cells, but prolonged exposure impairs cellular proliferation, underscoring the cell type- and exposure-dependent nature of its effects.

Our next experiments explored the induction of apoptotic markers following TBC exposure. At concentrations of 50 and 100 µM, TBC significantly increased the activity of caspase-3 and caspase-9 after both 24 and 48 h of exposure, while caspase-1 activity increased even at 10 µM TBC. Prior research has shown that exposure to 5–50 µM/kg of TBC in rat hippocampal cells results in decreased BCl2 activity and increased Bax and caspase-3 activity [17]. Furthermore, studies on SH-SY5Y cells exposed to 12.5–50 µM TBC also reported increased Bax levels and decreased Bcl-22 protein expression [16]. Similarly, Szychowski et al. (2021) observed increased caspase-3 activity in SH-SY5Y cells differentiated for 14 days at concentrations ranging from 1 nM to 100 µM [21] and Qi et al. (2022) identified mitochondrial pathway activation involving death receptors in TBC-exposed Hep-G2 cells [49].

Caspase-9, activated by apoptotic bodies, triggers a cascade reaction leading to apoptosis [50]. Notably, caspase-9 also promotes autophagy by maintaining mitochondrial homeostasis [51]. Our study suggests that both autophagy and apoptosis pathways may be activated in response to TBC exposure, with the predominant pathway potentially being dose-dependent. The activation of caspase-1 at 10 µM TBC in our study may also indicate the initiation of an inflammatory process. Supporting this, studies on primary mouse astrocytes exposed to 50 and 100 µM TBC demonstrated increased levels of inflammation markers (catalase, IL-1β and IL-1βR1 proteins) and decreased proliferation markers (Ki67) [19]. The inflammatory process is known to be a key factor in the development of neurodegenerative diseases [52, 53].

Based on the dose–response data for TBC cytotoxicity, we selected a dose of 10 µM, which reduced the metabolic activity of HT-22 cells in the resazurin reduction assay, for co-treatment experiments with CAY10464 (an AhR antagonist), GW9662 (a PPARγ antagonist) and honokiol (an NF-κB inhibitor). In our study, AhR protein expression decreased after 24 h exposure to 10 µM TBC but increased after 48 h with honokiol or honokiol + TBC, suggesting AhR involvement in longer-term hippocampal responses. This aligns with previous findings that AhR mediates TBC’s effects in SH-SY5Y cells, where CAY10464 reduced ROS production and increased cell viability [21].

The role of AhR in astrocyte-driven inflammation has been documented in experimental mouse models of multiple sclerosis [54]. Our results align with these findings, showing increased AhR expression after TBC and honokiol treatment. AhR’s anti-inflammatory effects may depend on interactions with other molecular pathways [55]. Although AhR activation can have adverse effects, as seen with TCDD in mice [56–58], our results showed increased PPARγ expression after 24 h in all groups, indicating potential anti-inflammatory and neuroprotective responses. PPARγ agonists are known to modulate oxidative stress and inflammation via interactions with Nrf2, CREB and HIF-1 and have demonstrated neuroprotective roles in various models, including TBBPA exposure [58–65]. Consistently, we observed a reduction in mTOR protein expression, suggesting activation of neuroprotective mechanisms, potentially via autophagy, in HT-22 cells [57].

In our study, TBC exposure increased PPARγ activity and involved the NF-κB pathway, indicating modulation of oxidative stress and inflammation. We observed that TBC reduced NLRP3 expression across all groups, while co-treatment with CAY10464, GW9662, or honokiol further suppressed NLRP3, suggesting a potential synergistic effect. IL-1β and IL-6 levels were elevated in co-treatment groups, though IL-1β was lower than with TBC alone after 48 h, reflecting nuanced inflammatory regulation. We also confirmed in our experiments that NF-κB regulates the production of key pro-inflammatory cytokines, including IL-1β, IL-6 and TNF-α, in a time-dependent manner, central to the initiation and amplification of the inflammatory response. These findings are consistent with NF-κB’s role in controlling NLRP3 transcription and inflammasome activity [66], suggesting that the observed decrease in NLRP3 may result from an overall suppression of the inflammatory response.

The NLRP3 inflammasome is critical for processing pro-IL-1β into active IL-1β, enhancing production of IL-6 and TNF-α [67], as observed in our study. Notably, increased phosphorylated p-IkBα after 48 h co-treatment with TBC and CAY10464, GW9662, or honokiol indicates NF-κB activation. Previous studies show that GW9662 can exert both anti- and pro-inflammatory effects depending on context [68, 69], while AhR activation or silencing modulates inflammation via NF-κB interactions, providing context-dependent pro- or anti-inflammatory effects [70–73]. Our results also revealed that micromolar TBC increased intracellular Ca^2^⁺, consistent with reports that dysregulated calcium homeostasis contributes to neurodegeneration, synaptic dysfunction and apoptosis [74–77]. Co-treatments influenced antioxidant responses, with CAY10464 + TBC and GW9662 + TBC increasing SOD1 expression, suggesting protective effects, while catalase and NQO1 decreased in some groups, indicating differential regulation. Elevated Ca^2^⁺ may activate Nrf2 signaling via Keap1 release, promoting transcription of antioxidant genes (HO-1, NQO1, SOD1/2, catalase) to neutralize ROS [68, 69]. Thus, calcium dysregulation can exacerbate oxidative stress and inflammation and compromise cellular defense mechanisms.

**Fig. 10:**
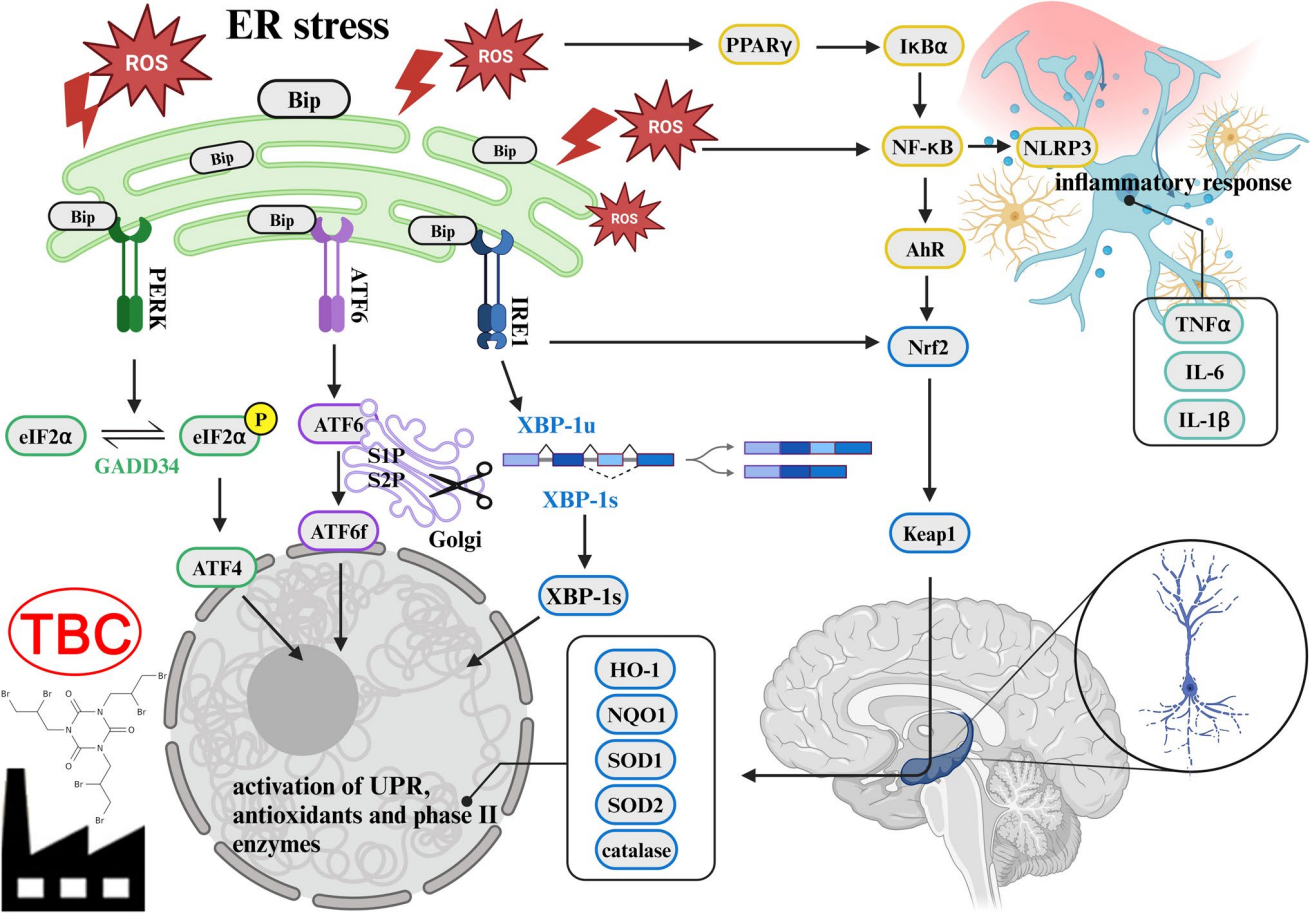
Proposed cell signaling mechanism controlled by TBC. Created with BioRend.com

Research on novel brominated flame retardants (NBFRs) and their effects on the nervous system is still limited. While some compounds, like TDCPP, TPhP, BEH-TEBP and EH-TBB, do not impair neuronal function at low concentrations [78], others, including TBBPA, BDE-209, DBDPE and tributyltin, activate NF-κB, increase ROS, induce ER stress and elevate pro-inflammatory cytokines such as TNFα, IL-1β and IL-6 [2, 78–85]. Consistently, in our study, TBC exposure triggered inflammatory responses in murine hippocampal HT-22 cells, including reduced metabolic activity; increased caspase-1, −3, −9 activity; elevated NF-κB; activation of the NLRP3 inflammasome; and increased IL-1β expression. TBC also dysregulated antioxidant responses (SOD1, catalase) and ER stress markers (Bip, IRE1, PERK, ATF6), with co-treatments (CAY10464, GW9662, honokiol) modulating these effects, suggesting potential protective or regulatory roles. Overall, our findings highlight the complex, time-dependent modulation of inflammatory, oxidative and ER stress pathways by TBC, supporting the use of hippocampal cells as a model for neurodegenerative disease research and providing insights into molecular mechanisms potentially relevant to human neurotoxicity (Fig. 10).

Despite providing novel insights into the effects of TBC on hippocampal cells, our study has several limitations. First, experiments were conducted in a single murine cell line (HT-22), which may not fully represent the complexity of the human nervous system. Second, exposure durations were relatively short and chronic effects of TBC remain to be investigated. Third, while we examined multiple pathways, including NF-κB, PPARγ, NLRP3, ER stress and oxidative stress, the exact molecular interactions and causal relationships require further mechanistic studies. Finally, in vitro conditions cannot fully replicate in vivo physiology, so additional animal or human-based studies are needed to confirm the relevance of these findings to neurodegenerative disease development.

In summary, our study demonstrates that TBC exposure modulates inflammatory, oxidative and ER stress pathways in murine hippocampal cells, involving PPARγ, NF-κB and NLRP3 signaling. Co-treatments with CAY10464, GW9662 and honokiol further influenced these responses, highlighting potential modulatory or protective effects. These findings provide insights into the molecular mechanisms underlying TBC-induced neurotoxicity and support the use of hippocampal cells as a model for studying environmental neurotoxicants and their implications for neurodegenerative disease.

## Supplementary Information

Below is the link to the electronic supplementary material.

ESM 1 (PDF 244 KB)

## Data Availability

The datasets used during the current study are available from the corresponding author on reasonable request.

## Abbreviations

AhR: Aryl hydrocarbon receptor
ATF6: Activating transcription factor 6
Bax: Bcl-2-associated X-protein
BCA: Bicinchonic acid
Bcl2: B cell lymphoma 2
BEH-TEBP: Bis(2-ethylhexyl) tetrabromophthalate
Bip: Binding immunoglobulin protein
BSA: Bovine serum albumin
CHAPS: 3-[(3-Cholamidopropyl)dimethylamino]-1-propanesulfonate hydrate
DBDPE: Decabromodiphenyl ethane
DMEM: Dulbecco’s Modified Eagle Medium
DTT: Dithiothreitol
EDTA: Ethylenediaminetetraacetic acid
EH-TBB: 2-Ethylhexyl-2,3,4,5-tetrabrombenzoat
eIF2α: Eukaryotic initiation factor 2 alpha
GAPDH: Glyceraldehyde-3-phosphate dehydrogenase
HO-1: Heme oxygenase
H33342: Hoechst 33,342
HEPES: Hydroxyethyl piperazine ethane sulfonic acid
HIF1: Hypoxia inducible factor
HUVEC: Human umbilical vein endothelial cells
IL-1β: Interleukin-1 β
IL-6: Interleukin-6
INT: 2-P-Iodophenyl-3-p-nitrophenyl-5-phenyl tetrazolium chloride
IRE1α: The serine/threonine-protein kinase/endoribonuclease inositol-requiring enzyme 1 α
IκBα: Nuclear factor of kappa light polypeptide gene enhancer in B cell inhibitor alpha
Ki67: Marker of proliferation Kiel 67
LDH: Lactate dehydrogenase
Keap1: Kelch-like ECH-associated protein 1
LPS: Lipopolysaccharide
MPMS: 1-Methoxyphenazine methosulfate
MS: Multiple sclerosis
mTOR: Mammalian target of rapamycin
NAD: *β*-Nicotinamide adenine
NBFRs: New brominated flame retardants
NF-κB: Nuclear factor kappa light chain enhancer of activated B cells
NLRP3: Nod-like receptor protein 3
NQO1: NAD(P)H dehydrogenase (quinone)
Nrf2: Nuclear factor erythroid 2-related factor 2
pIKBα: Phosphorylated IκBα
PERK: Protein kinase R (PKR)-like endoplasmic reticulum kinase
PPARγ: Peroxisome proliferator-activated receptor gamma
ROS: Reactive oxygen species
SDS: Sodium dodecyl sulfate
SOD: Superoxide dismutase 1
TBBPA: Tetrabromobisphenol A
TBC: Tris(2,3-dibromopropyl)isocyanurate
TDCPP: Tris(1,3-dichloroisopropyl)phosphate
TEMED: N,N,N′,N′-tetramethylethylenediamine
TNFα: Tumor necrosis factor α
TPhP: Triphenyl phosphate
XBP1: Xbox binding protein
