# Supplementary material for: Targeting Tris(2,3-dibromopropyl) Isocyanurate-Induced Inflammation in Hippocampal Neurons In Vitro: Mechanistic Insights and Implications for Neurodegenerative Disease Prevention
Source: Mol Neurobiol. 2025 Nov 19;63(1):84. doi: 10.1007/s12035-025-05301-w (PMC12627138; doi:10.1007/s12035-025-05301-w)

48h

mTOR

Control TBC CAY10464 TBC+CAY10464 GW9662 TBC+GW9662 Honokiol TBC+Honokiol

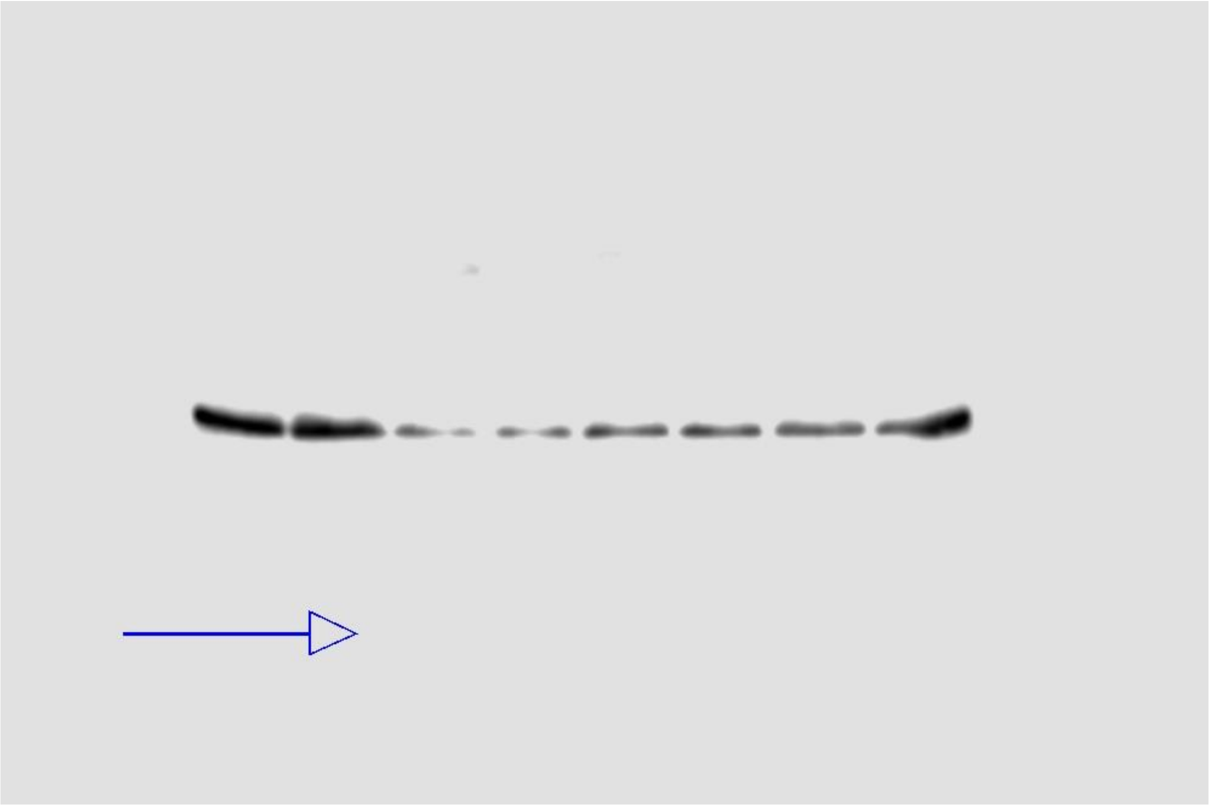

NFKB

Control TBC CAY10464 TBC+CAY10464 GW9662 TBC+GW9662 Honokiol TBC+Honokiol

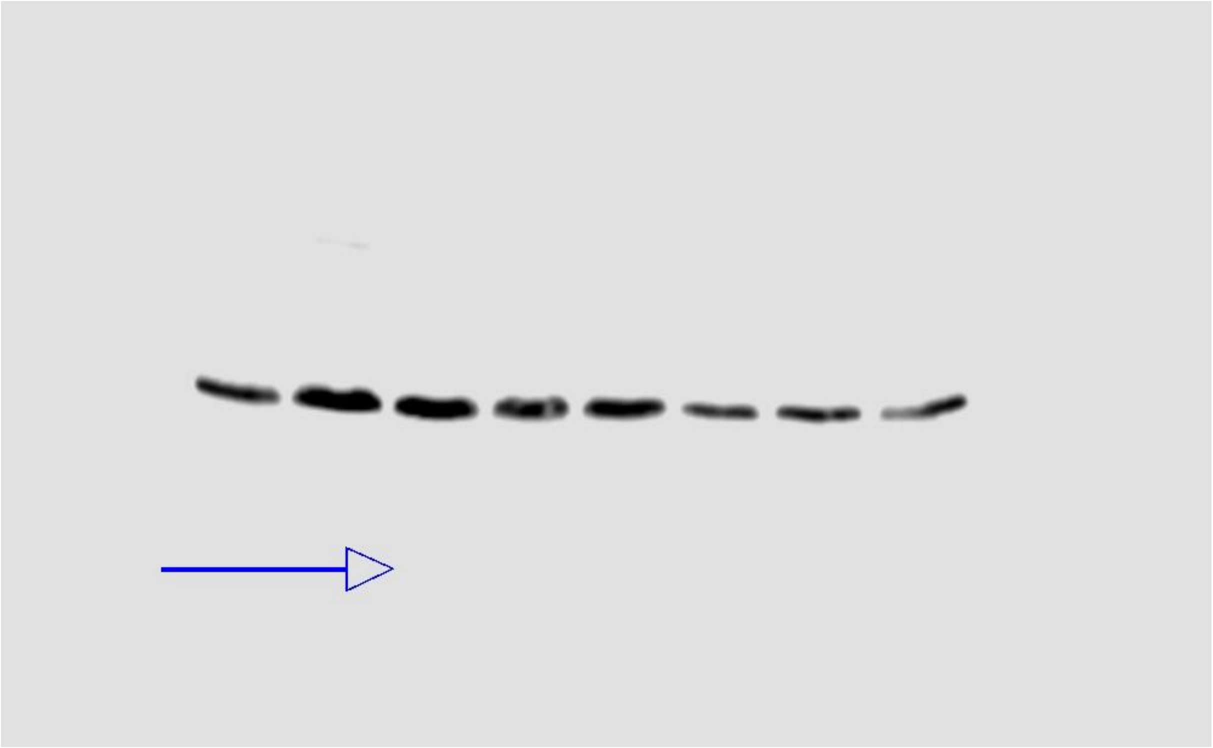

p-IKBa

Control TBC CAY10464 TBC+CAY10464 GW9662 TBC+GW9662 Honokiol TBC+Honokiol

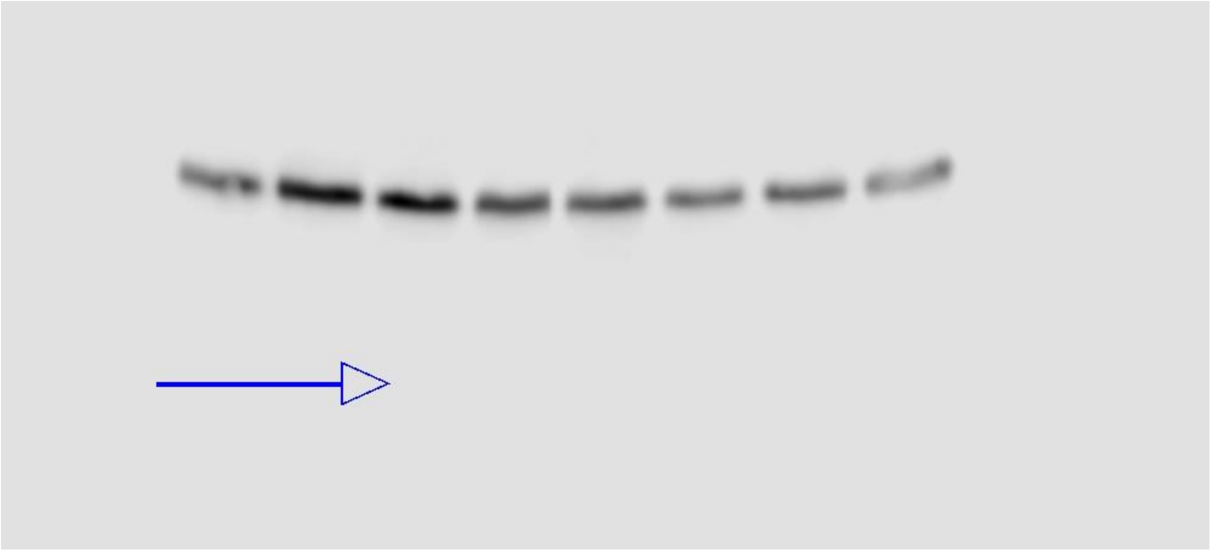

GAPDH

Control TBC CAY10464 TBC+CAY10464 GW9662 TBC+GW9662 Honokiol TBC+Honokiol

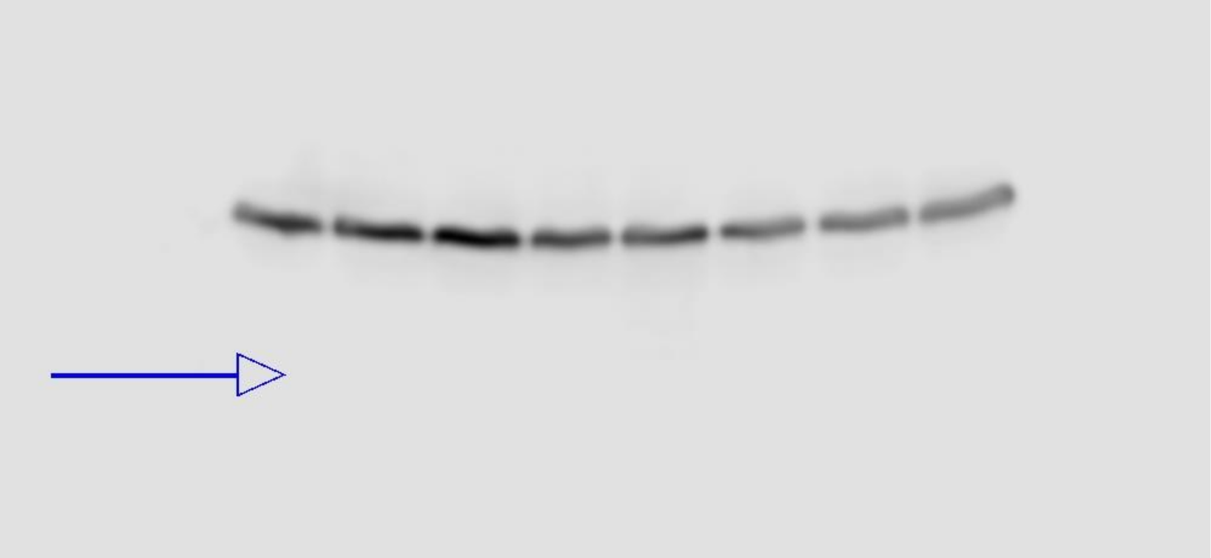

Ahr

Control TBC CAY10464 TBC+CAY10464 GW9662 TBC+GW9662 Honokiol TBC+Honokiol

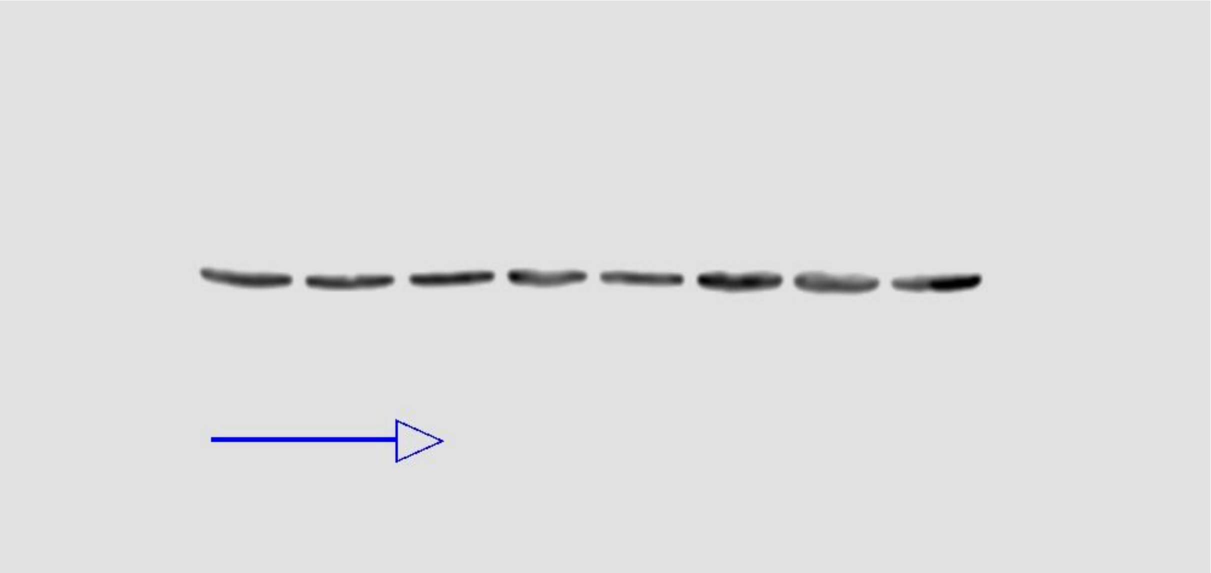

PPARγ

Control TBC CAY10464 TBC+CAY10464 GW9662 TBC+GW9662 Honokiol TBC+Honokiol

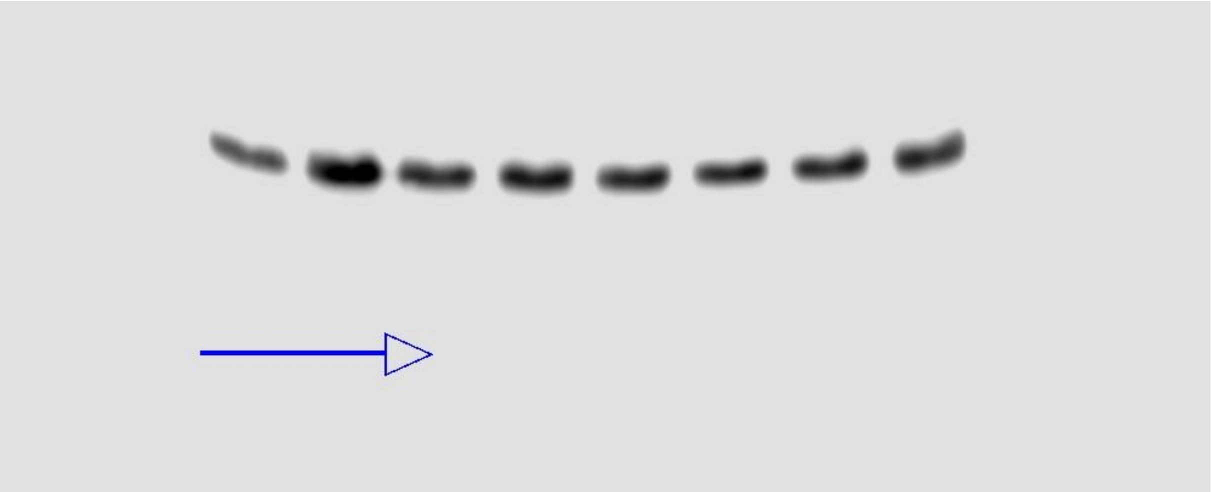

IKBa

Control TBC CAY10464 TBC+CAY10464 GW9662 TBC+GW9662 Honokiol TBC+Honokiol

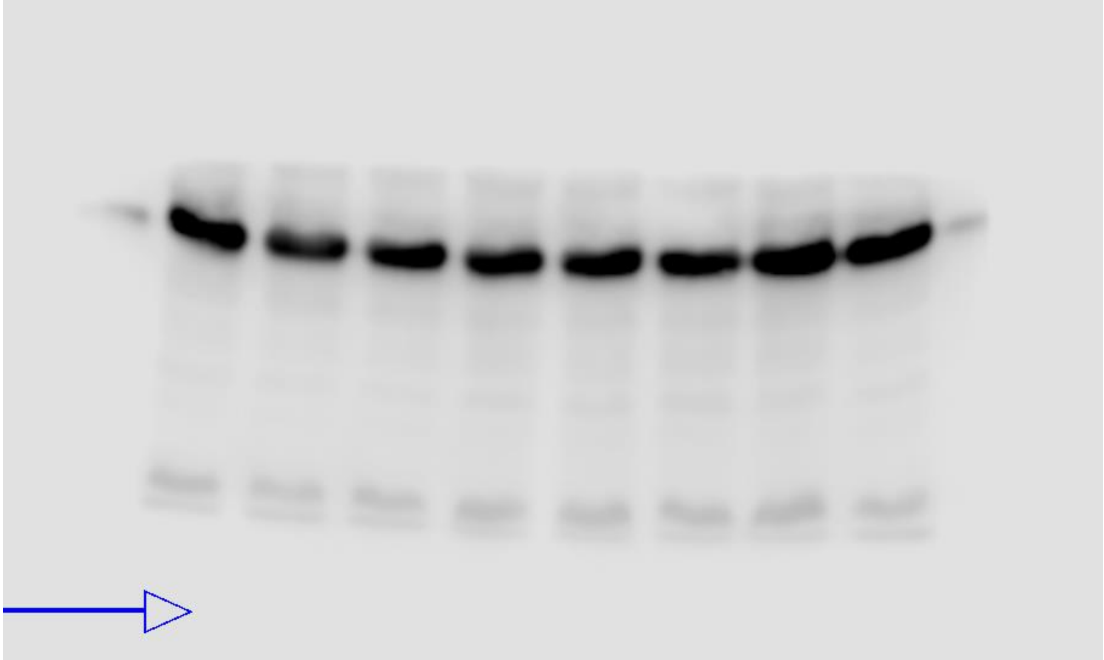

GAPDH

Control TBC CAY10464 TBC+CAY10464 GW9662 TBC+GW9662 Honokiol TBC+Honokiol

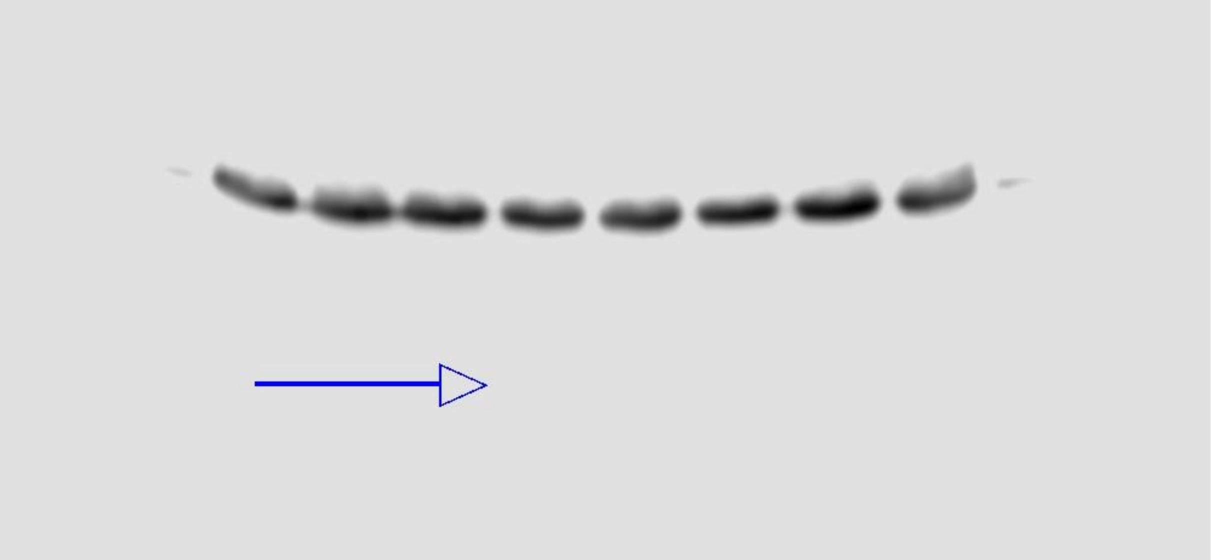

24h

mTOR

Control TBC CAY10464 TBC+CAY10464 GW9662 TBC+GW9662 Honokiol TBC+Honokiol

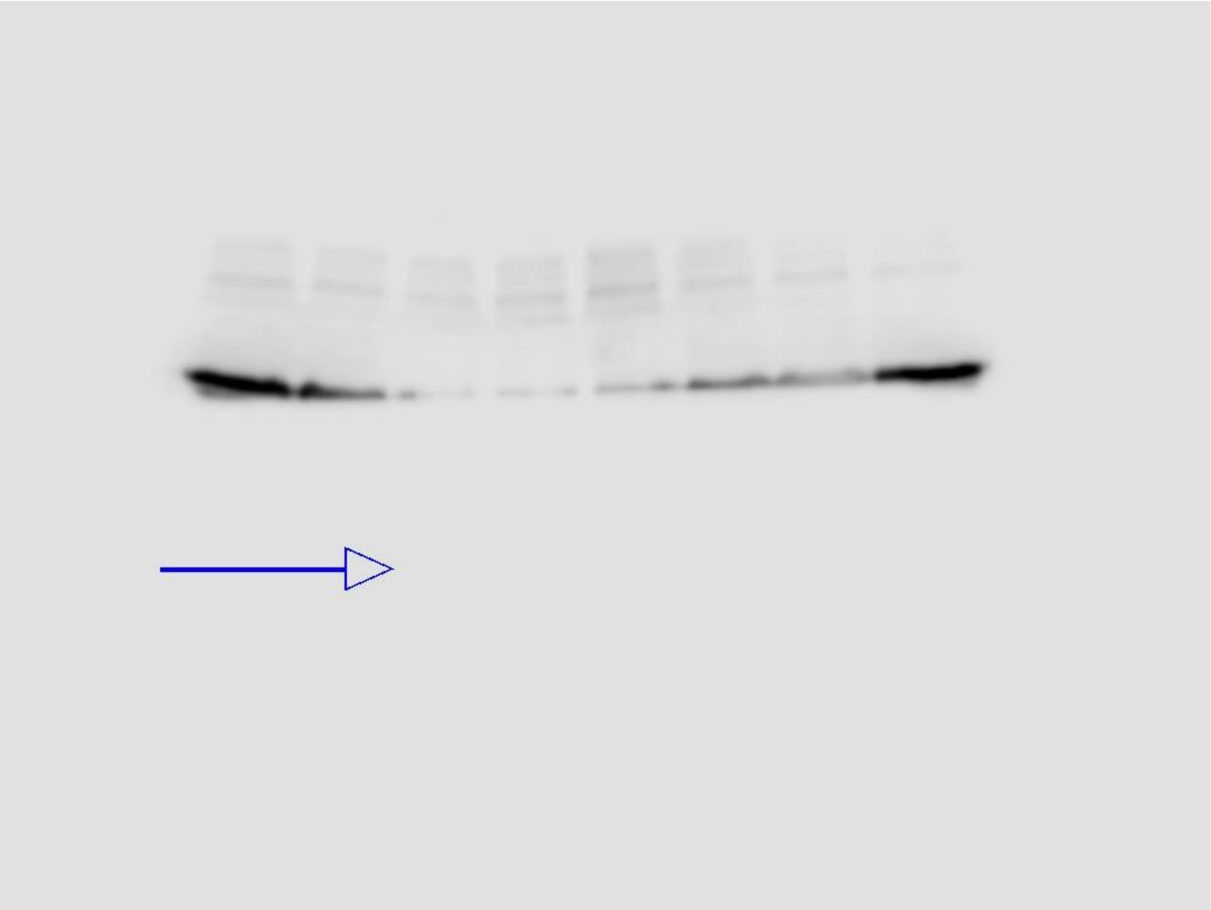

NF-KB

Control TBC CAY10464 TBC+CAY10464 GW9662 TBC+GW9662 Honokiol TBC+Honokiol

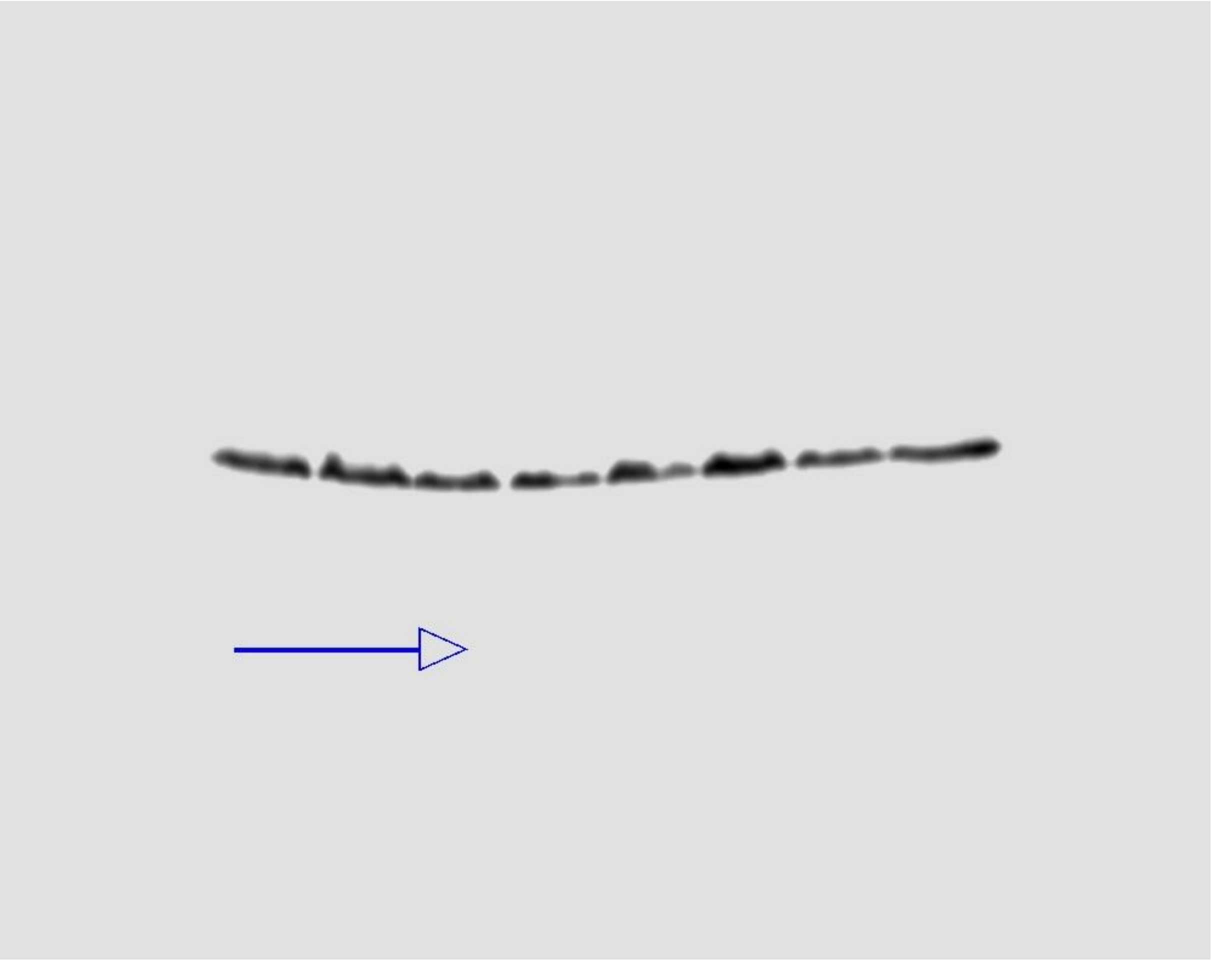

p-IKBa

Control TBC CAY10464 TBC+CAY10464 GW9662 TBC+GW9662 Honokiol TBC+Honokiol

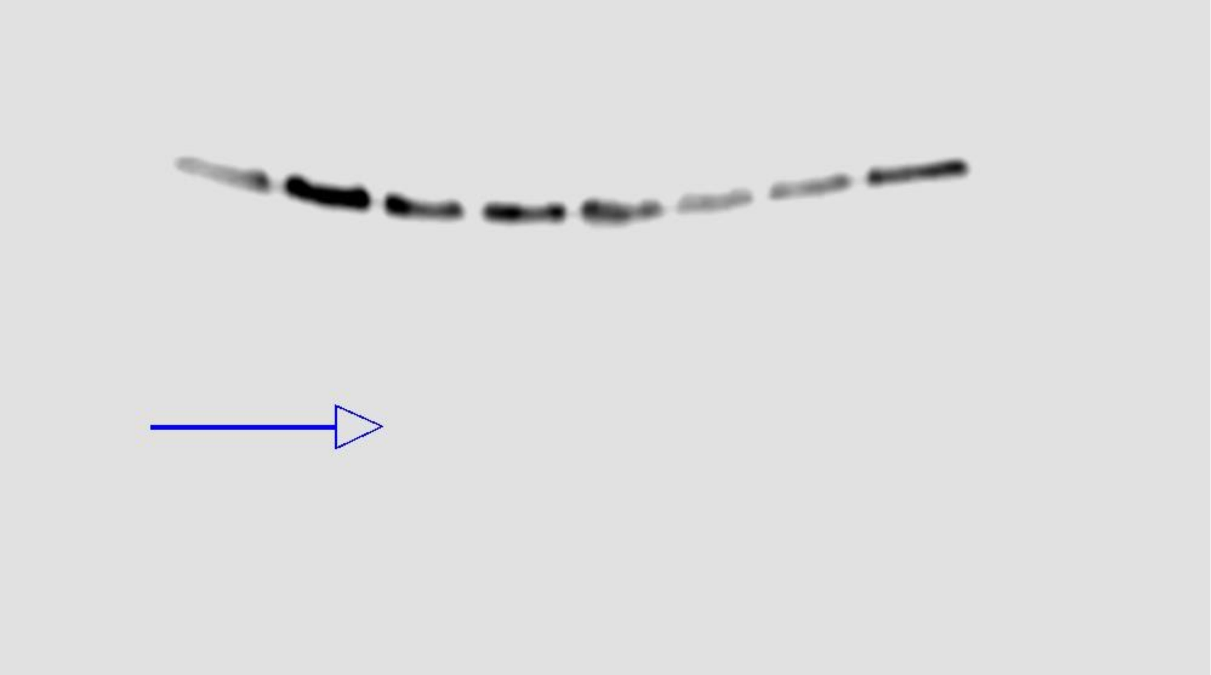

GAPDH

Control TBC CAY10464 TBC+CAY10464 GW9662 TBC+GW9662 Honokiol TBC+Honokiol

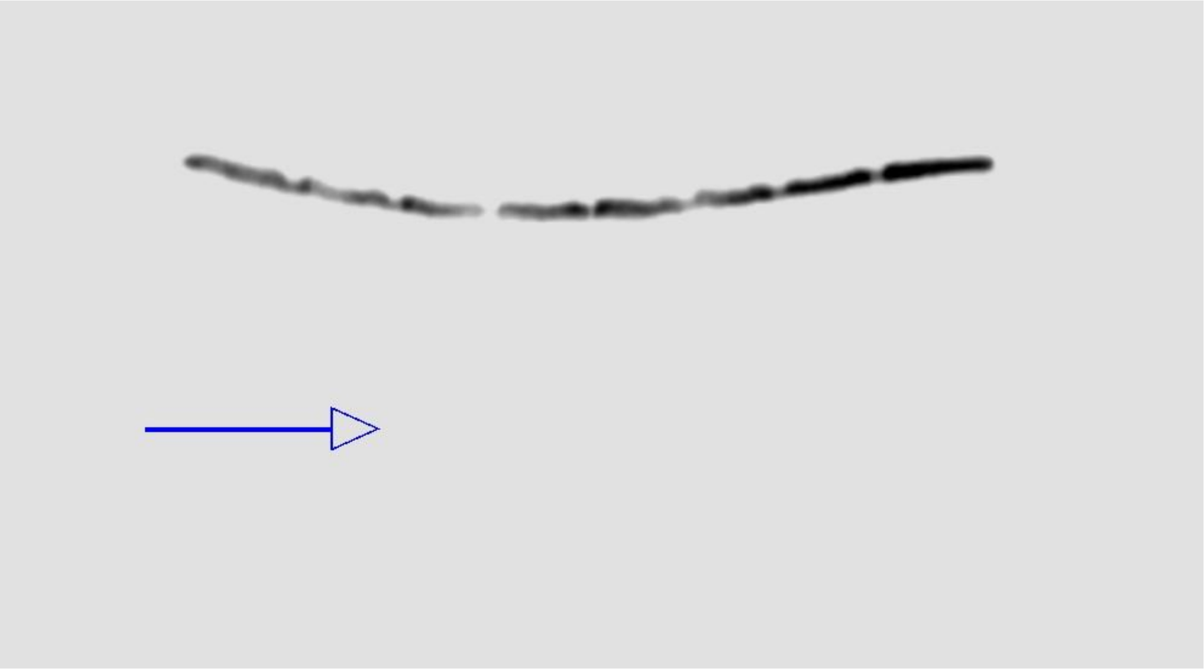

Ahr

Control TBC CAY10464 TBC+CAY10464 GW9662 TBC+GW9662 Honokiol TBC+Honokiol

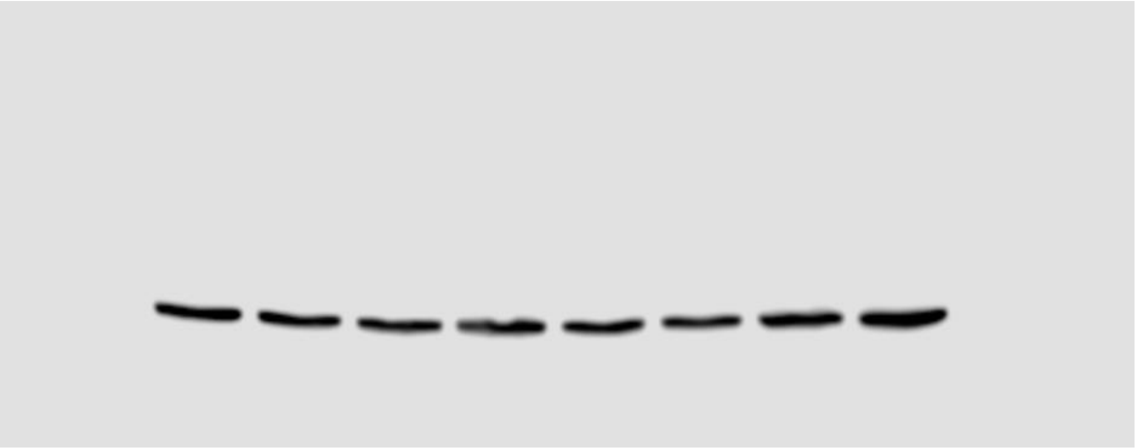

PPARγ

Control TBC CAY10464 TBC+CAY10464 GW9662 TBC+GW9662 Honokiol TBC+Honokiol

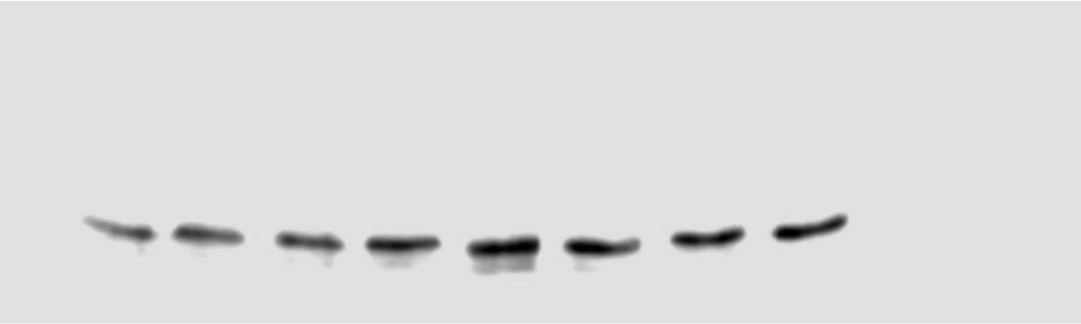

IKBa

Control TBC CAY10464 TBC+CAY10464 GW9662 TBC+GW9662 Honokiol TBC+Honokiol

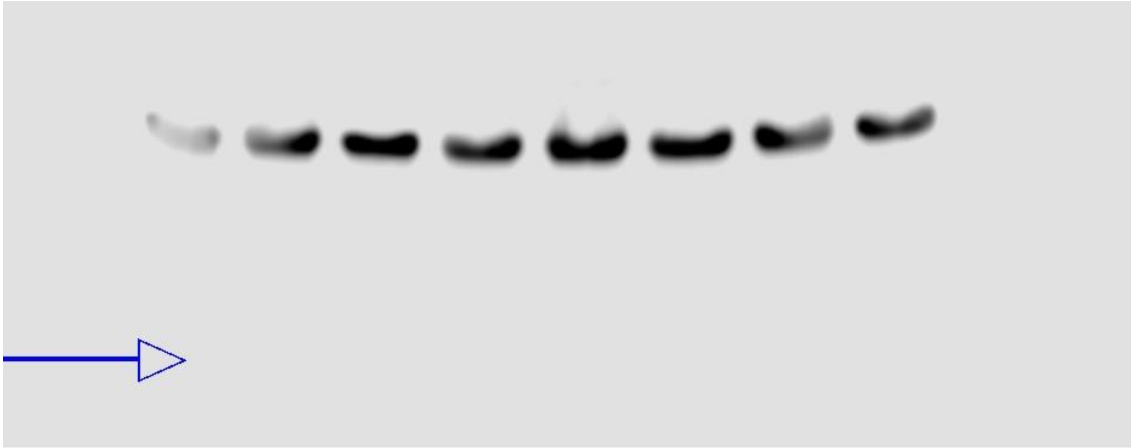

GAPDH

Control TBC CAY10464 TBC+CAY10464 GW9662 TBC+GW9662 Honokiol TBC+Honokiol

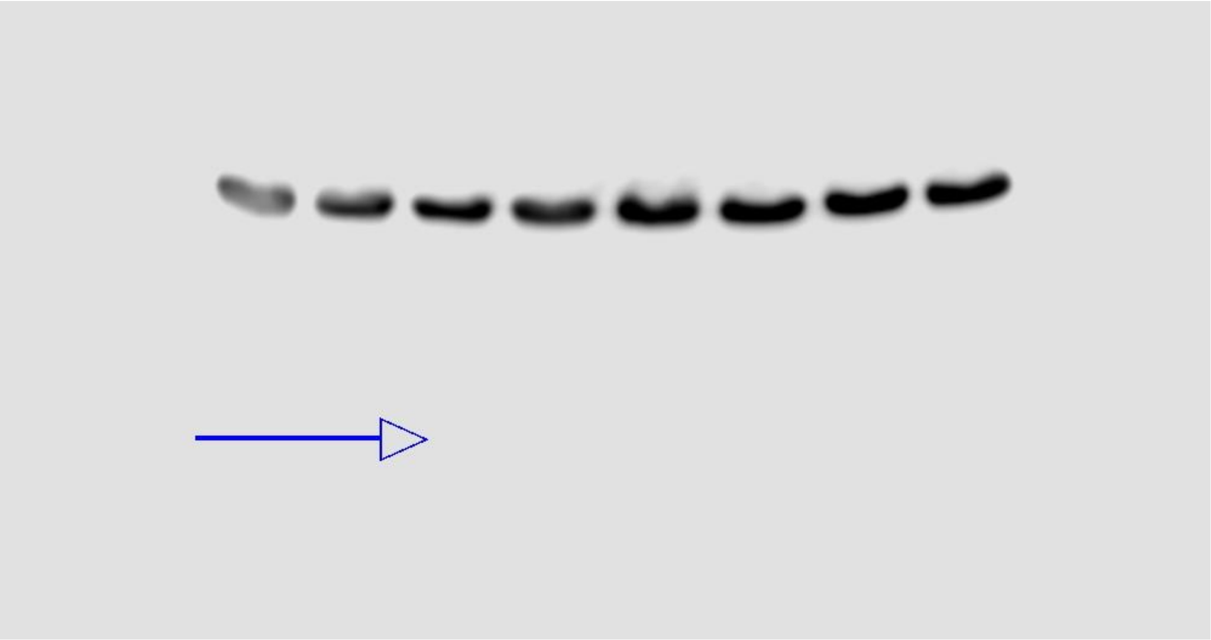

Supplement: Supplementary file 1 — (PDF 244 KB) [file 12035_2025_5301_MOESM1_ESM.pdf]
